# Exploring the Potential and Advancements of Circular RNA Therapeutics

**DOI:** 10.1002/EXP.20240044

**Published:** 2025-05-01

**Authors:** Lei Wang, Lianqing Wang, Chunbo Dong, Jialong Liu, Guoxin Cui, Shan Gao, Zhida Liu

**Affiliations:** ^1^ Shanxi Academy of Advanced Research and Innovation Taiyuan China; ^2^ MOE Key Laboratory of Coal Environmental Pathogenicity and Prevention Shanxi Medical University Taiyuan China; ^3^ Department of Biochemistry and Molecular Biology Shanxi Key Laboratory of Birth Defect and Cell Regeneration College of Basic Medical Sciences Shanxi Medical University Taiyuan China; ^4^ Center of Translational Medicine Zibo Central Hospital Zibo China; ^5^ College of Veterinary Medicine Shanxi Agricultural University Jinzhong China; ^6^ Red Sea Research Center Biological and Environmental Science and Engineering Division King Abdullah University of Science and Technology (KAUST) Thuwal Saudi Arabia; ^7^ Zhongda Hospital School of Life Sciences and Technology Advanced Institute for Life and Health Southeast University Nanjing China

**Keywords:** back‐splicing, circRNA, IRES, mRNA, ribozyme, therapeutics

## Abstract

Messenger RNA (mRNA) technology is revolutionizing the pharmaceutical industry owing to its superior safety profile, manufacturing capabilities, and potential applications in previously undruggable therapeutic targets. In addition to linear mRNA, such as conventional mRNA, self‐amplifying mRNA, and trans‐amplifying mRNA, circular mRNA has emerged as a promising candidate. Circular RNA (circRNA) is a class of single‐stranded RNA with a covalently closed loop structure that offers enhanced stability compared to linear RNA by resisting degradation from RNases. Recent studies have revolutionized our understanding of their biological functions, surpassing the notion that they are merely byproducts of aberrant splicing events. Given the remarkable success achieved in cancer and SARS‐CoV‐2/monkeypox virus (MPXV) vaccines, circRNA is being intensively investigated for gene and cell therapies. In this review, we provide an overview of circRNA biogenesis mechanisms in vivo, along with synthesis strategies in vitro, while discussing translation regulation mechanisms and quality control processes involved in circRNA production. Furthermore, we explore the potential application scenarios for circRNAs.

## Graphical abstract

Given the remarkable advantages in terms of stability, sustained expression profile, safety, wide range of druggable targets, scalable and cost‐effective manufacturing capabilities, circRNA is currently undergoing intensive investigation for various therapeutic applications such as vaccines, protein replacement, genetic disease treatment, gene therapy, and cell therapy. This review provides an overview of circRNA biogenesis, synthesis strategies, translation regulation, purification, quality control, and potential therapeutic applications. Additionally included are discussions on potential obstacles, challenges, and future perspectives in the development of circRNA therapeutics.

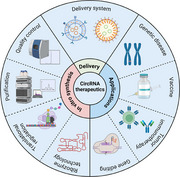

## 1 Introduction

Over the past few decades, with the rapid advancements and progress in molecular biology, RNA technology, nanotechnology, and other related techniques, mRNA‐based therapeutics have revolutionized the pharmaceutical industry. This is primarily due to their ability to harness the human body as a factory for producing a wide range of therapeutic proteins or peptides that target various diseases, including previously undruggable targets. Furthermore, mRNA can be efficiently produced through IVT, which is cost‐effective, faster, and more flexible in design. Another significant advantage of mRNA therapeutics is that they do not require nuclear entry for functionality and pose no potential risk of genomic insertion. Given these inherent benefits, mRNA emerges as an ideal platform not only for vaccine development during large‐scale outbreaks of emerging infectious diseases (such as the COVID‐19 pandemic), but also for readily available off‐the‐shelf therapeutics and even personalized medications [1]. However, there are still technical challenges that need to be addressed regarding the stability, immunogenicity, translation efficiency, and delivery system of mRNA [2]. Extensive efforts have been made to overcome these pivotal issues. Nucleoside‐chemical modification and sequence optimization techniques have shown promise in reducing immunogenicity and improving translation efficiency [3]. Numerous lipid nanoparticle (LNP)‐based packaging systems have been developed to enhance the stability and enable effective organ/cell selective delivery of mRNA molecules [4]. What is more, novel types of mRNA, such as self‐amplifying mRNA [5], trans‐amplifying mRNA [6], and circular mRNA [7], were developed to increase and prolong therapeutic protein production. Among these, circRNA, also termed mRNA 2.0, is increasingly favored owing to its superior stability, sustained expression profile, and low immunogenicity [8].

CircRNAs are biologically active single‐stranded RNA molecules that adopt closed‐loop structures and lack 5' caps and 3' polyadenylated tails, distinguishing them from linear mRNAs [9]. The discovery of circRNAs dates back to the 1970s when they were first identified in plant viroids [10]. Over the subsequent decades, extensive research has dispelled the misconception that circRNAs are mere byproducts of aberrant RNA splicing [11]. Meanwhile, the dominant formation mechanism of circRNAs has been elucidated as back‐splicing, wherein a downstream 5' splice‐donor site attacks an upstream 3' splice‐acceptor site and generates the 3',5' phosphodiester bond [12]. In recent years, advancements in high‐throughput RNA‐seq technology have unveiled the abundance and diversity of circRNAs [13]. The majority of circRNAs are localized in the cytoplasm, with only a minority found in the nucleus [14]. Numerous studies have provided compelling evidence that circRNAs function as microRNA (miRNA) sponges, protein sponges or scaffolds, regulators of gene expression, and biomarkers in cells [15]. Consequently, native circRNAs themselves can serve as potential targets for disease treatment [16]. However, our current understanding of circRNA is merely scratching the surface, and a comprehensive elucidation of their biological functions and mechanisms remains quite distant.

By 2017, the identification of two protein‐coding circRNAs initiated investigations into the protein translation functions of circRNAs [17]. Furthermore, several circRNA databases were established to document circRNA information [18], and algorithms were developed to explore their translational potential [19]. Despite lacking a 5' cap and 3' polyadenylated tail essential for mRNA translation, circRNAs can initiate translation through cap‐independent mechanisms [12a]. Consequently, there is an expectation that mRNA‐based therapies could be translated into circRNA‐based therapies. In 2018, the circRNAs synthesized via the permuted intron‐exon (PIE) strategy in vitro were demonstrated to possess a strong and durable translation ability in eukaryotic cells [20]. Similar to linear mRNA, circRNAs can also be efficiently delivered and translated both in vitro and in vivo when packaged with LNP [7, 21]. The successful development of circRNA‐based vaccines against SARS‐CoV‐2, MPXV, and malignant tumors has marked the great potential for circRNA therapeutics, especially in the pharmaceutical industry [7, 22]. The circRNA therapeutics not only inherit the advantages of mRNA‐based therapeutics, but also display better stability and durable expression than mRNAs in cells due to they are more resistant to RNases, which is attributed to their covalently closed loop structure. Moreover, in vitro preparation procedures for circRNAs are characterized by simplicity and cost‐effectiveness, as they obviate the need for modification processes involving 5' cap and 3' polyadenylated tail. However, numerous challenges persist in the development of synthetic circRNAs as therapeutic agents, including enhancing the translational efficiency of circRNAs and establishing large‐scale manufacturing processes for producing highly purified circRNAs.

This review will primarily focus on the current advancements in circRNA research, encompassing biogenesis, synthesis strategies in vitro, translation regulation, quality control, and potential therapeutic applications. These comprehensive insights are expected to contribute significantly toward the development of circRNAs as promising therapeutics.

## 2 Biogenesis of circRNAs

CircRNAs exhibit diverse functional roles in various biological processes and can be classified into three primary groups based on their components: exon‐intron circRNAs (EIcircRNAs), exonic circRNAs (EcircRNAs), and intron‐derived circRNAs (IcircRNAs) [23]. The group of intron‐derived circRNA encompasses both intronic circRNAs (CiRNAs) derived from pre‐mRNA splicing and tRNA intronic circRNAs (TricRNAs) [24]. Despite initially being considered as byproducts of aberrant RNA splicing, extensive research conducted over the past few decades has refuted this misconception and proposed their mechanisms of formation [11].

### 2.1 Back‐Splicing Model

The available research indicates that the majority of circRNAs in eukaryotes are generated through back‐splicing of exonic and/or intronic sequences from pre‐mRNA, which competes with canonical mRNA splicing [8, 23, 25]. Despite being less efficient than linear splicing [26], circRNAs can still accumulate in cells due to their resistance to RNases. It is important to note that back‐splicing differs from canonical splicing, which produces linear mRNA. Initially, DNA is transcribed into primary transcripts containing both exons and introns. During the canonical splicing process, introns are excised while exons are ligated together to form a mature mRNA molecule [27]. However, in the case of circRNAs, back‐splicing involves one or more exons. This process occurs when a downstream splice donor site joins with an upstream splice acceptor site, resulting in the formation of a covalently closed circRNA structure with a 3',5' phosphodiester bond at the back‐splicing junction site [26, 28]. The presence of specific *cis*‐acting elements within introns flanking the back‐spliced exons is crucial for RNA circularization. These elements can form a hairpin structure through base‐pairing, bringing the downstream 5' splice site and the upstream 3' splice site into close proximity [29]. Additionally, certain RNA‐binding proteins (RBPs) recognize and bind to specific motifs within flanking introns, facilitating the interaction between the two splice sites [30]. In some cases, EIcircRNA generated through back‐splicing can exclude the intron from the final circRNA product via canonical splicing, resulting in EcircRNA synthesis [26a].

### 2.2 Non‐Back‐Splicing Model

**FIGURE 1:**
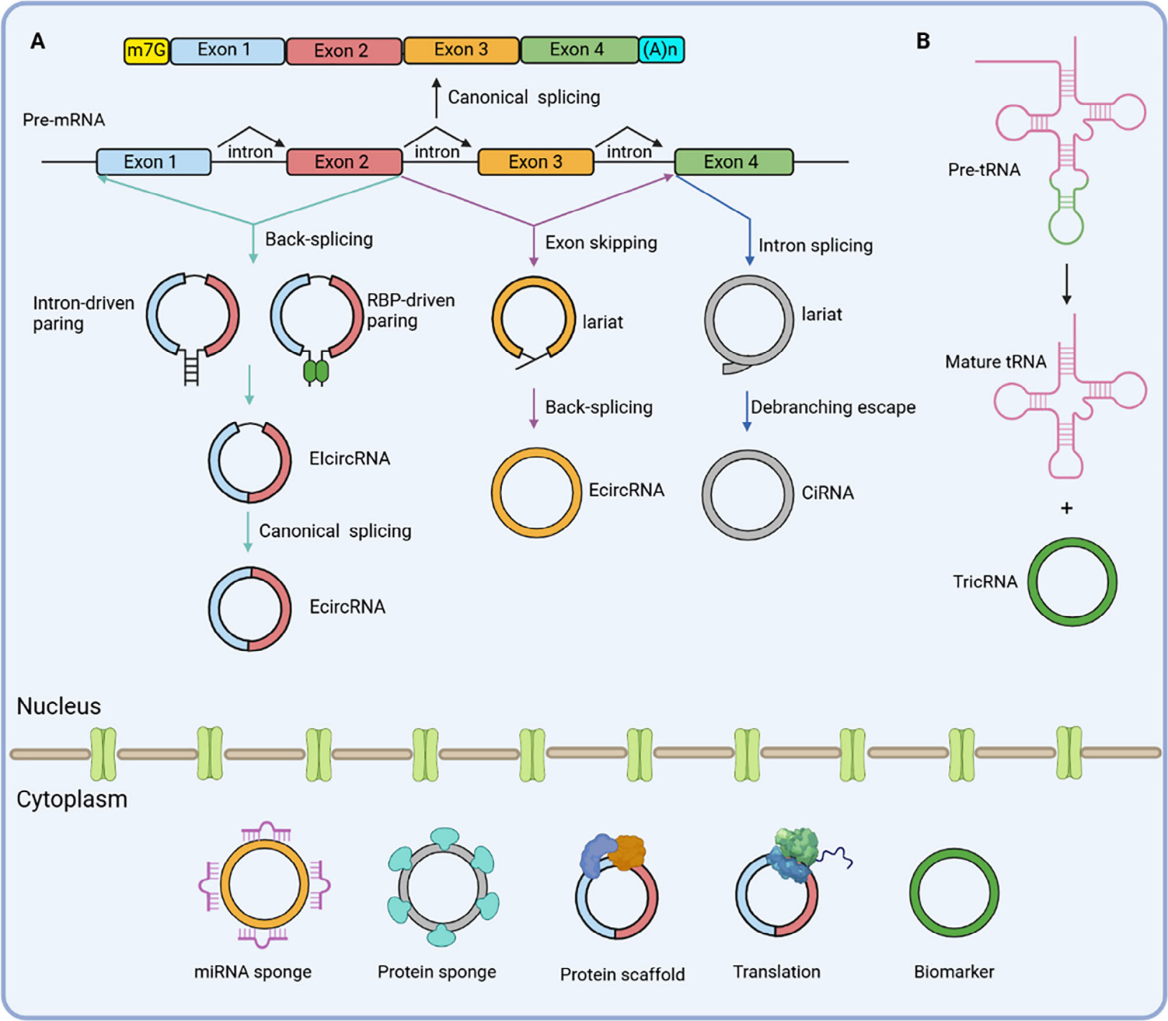
Models of circRNA biogenesis. (A) Pre‐mRNA can undergo inefficient back‐splicing, resulting in the generation of circRNAs. In the back‐splicing model, a hairpin structure can be organized either through base‐pairing or with the assistance of RBPs, bringing two splice sites close proximity. The intron sequences can be excluded or retained in the final RNA products, generating EcircRNAs or EIcircRNAs, respectively. Alternatively, the lariat‐driven model involves the formation of a lariat structure via canonical splicing during both exon‐skipping and intron splicing of pre‐mRNA. In exon‐skipping events, the lariat structure further undergoes internal back‐splicing to generate an EcircRNA. In the case of intron splicing, the lariat structure escapes debranching and is trimmed to produce CiRNAs. (B) Pre‐tRNA containing an intron is cleaved into the exon and intron halves. The exon halves and intron halves are then joined together to form a mature tRNA and a TricRNA, respectively.

Another mechanism for circRNAs biogenesis is the lariat‐driven model, which occurs during exon‐skipping events or intron splicing of pre‐mRNAs. In exon‐skipping events, specific exonic and intronic sequences are excised from pre‐mRNA by canonical splicing, resulting in the formation of a lariat structure. Subsequently, internal back‐splicing takes place within the lariat structure, leading to the removal of intronic sequences and the generation of EcircRNAs [26, 31]. Similarly, during intron splicing of pre‐mRNA, an intronic lariat escapes debranching, and its 3' tail downstream branch point site is trimmed to generate CiRNAs (Figure 1A) [24, 32].

Apart from circRNAs generated through spliceosome‐dependent processes from pre‐mRNA, a small subset of intron‐derived circRNAs originates from pre‐tRNA, whose post‐transcriptional processing occurs independently of the spliceosome. In certain cases of pre‐tRNA maturation, the tRNA splicing endonuclease complex cleaves the intron of pre‐tRNA and ligates the intron termini together to form a tRNA intronic circRNA known as TricRNA (Figure 1B) [33]. Additionally, other types of intronic circRNAs can be produced by self‐splicing intron systems, such as group I and group II introns [34]. Moreover, circRNAs are found in viroids, viroid‐like satellite RNAs, and hepatitis delta virus (HDV). These specific circRNAs originate from a rolling‐circle replication process where a circular template is utilized for reiterative transcription followed by cleavage of the produced oligomeric RNA molecule into monomeric RNA units that subsequently ligate to form circRNA structures [35].

Following their formation in the nucleus, a substantial proportion of circRNAs undergo transportation into the cytoplasm to fulfill diverse physiological functions (Figure 1) [23]. Consequently, these inherent mechanisms governing circRNA generation are currently adapted and employed for both in vitro and in vivo production of circRNAs, facilitating research endeavors and potential applications.

## 3 Strategies for circRNA Synthesis

Due to the diverse physiological functions in vivo, circRNAs have been deliberately designed and synthesized in vitro or in vivo as therapeutic agents for disease treatment and prevention. However, the current in vivo production of circRNAs still relies on plasmids as transcriptional templates, which poses a risk of integration into the genome upon delivery into cells [33, 36]. Therefore, in vitro synthesis of circRNAs is the preferred method in circRNA therapeutics. Here, we aim to summarize the strategies for in vitro circRNA synthesis. For comprehensive information on in vivo strategies for circRNA synthesis, we recommend referring to the excellent content reviewed by Obi et al. [37].

To synthesize circRNA in vitro, a linear RNA precursor is indispensable. Chemical synthesis and enzymatic strategies are the two primary methods employed for the preparation of linear RNA precursor [37]. However, chemical synthesis can only produce small RNA and requires costly purification, making it less preferable. On the other hand, the enzymatic strategy is favored for synthesizing the linear RNA precursors [38]. In this approach, IVT is performed using the DNA template, reaction buffer, and phage RNA polymerase. T7 RNA polymerase, being the most used phage RNA polymerase for IVT reactions, facilitates more efficient synthesis of longer RNA molecules. Various methods have been developed for ligating linear RNA precursors in vitro, including chemical ligation, enzymatic ligation, and ribozyme‐based approaches. Each technique possesses distinct characteristics, offering researchers a range of options to ligate linear RNA precursors and generate circRNAs for diverse research applications efficiently and accurately.

### 3.1 Chemical Ligation

**FIGURE 2:**
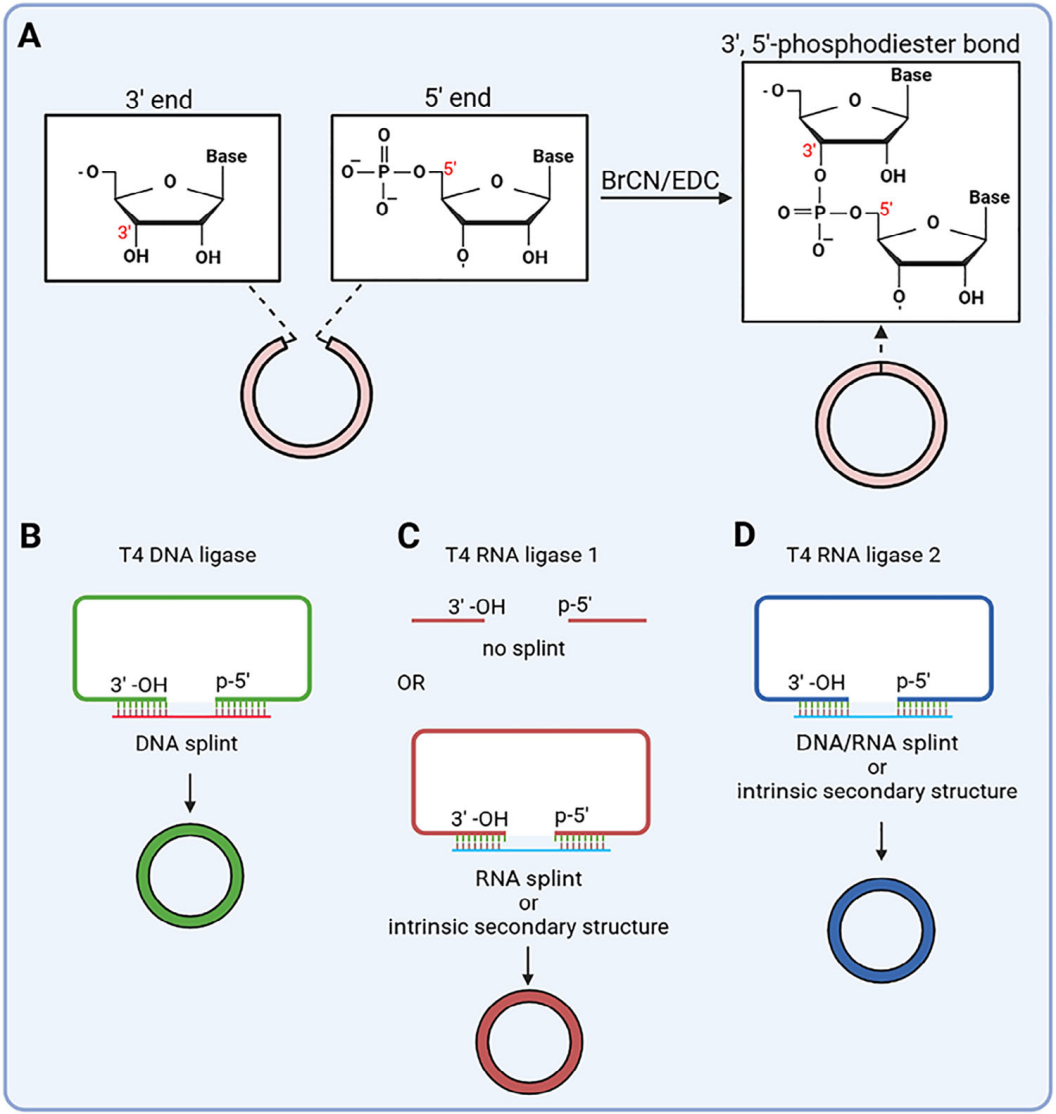
RNA circularization through chemical and enzymatic ligation. (A) Chemical ligation involves the joining of distal ends of the linear RNA precursor using the chemical reagent BrCN or EDC. (B–D) Three T4 ligation enzymes are employed for ssRNA circularization. T4 DNA ligase exhibits proficiency in ligating nicks in DNA/RNA hybrids (B). T4 RNA ligase 1 catalyzes end ligation of ssRNA, forming a covalent 3',5'‐phosphodiester bond either with or without a splint composed of a programmatic RNA. In certain instances, the intrinsic secondary structure can obviate the necessity for a splint by optimizing the ligation end sequences, thereby facilitating RNA circularization (C). However, T4 RNA ligase 2 demonstrates enhanced activity in joining RNA nicks with double‐stranded structures rather than ssRNA ends. Consequently, successful RNA circularization mediated by T4 RNA ligase 2 typically necessitates either the assistance of a DNA or RNA splint or the design of internal secondary structures at reaction ends of ssRNA to bring the 5'‐monophosphate and 3'‐OH into proximity (D).

The chemical ligation of 5' and 3' ends from a single‐stranded RNA (ssRNA) to generate circRNA is achieved through the utilization of cyanogen bromide (BrCN) or 1‐ethyl‐3‐(3'‐dimethylaminopropyl) carbodiimide (EDC) (Figure 2A) [39]. However, this approach presents certain challenges, including low ligating efficiency and significant side reactions. The competition between intermolecular and intramolecular reactions of RNA often results in the formation of tandem linear RNA products [40]. Additionally, chemical ligation generates 2',5'‐phosphodiester bonds instead of the natural 3',5'‐phosphodiester bonds, leading to a heterogeneous mixture of circRNAs [34]. Despite extensive efforts to address these limitations, chemical ligation remains less competitive compared to alternative circularization methods, such as enzymatic ligation and the ribozyme method [34]. Furthermore, the utilization of chemical reagents in the strategy of chemical ligation raises biosafety concerns and presents technological challenges for circRNA purification. Consequently, it is not favored for the in vitro synthesis of circRNA.

### 3.2 Enzymatic Ligation

To date, several enzymatic ligation strategies have been developed for the in vitro circularization of linear RNA precursor. These strategies employ three enzymes (T4 DNA ligase, T4 RNA ligase 1, and T4 RNA ligase 2) derived from the bacteriophage T4 to catalyze ligation reactions (Figure 2B–D). The enzymatic process involves catalyzing the formation of a 3',5'‐phosphodiester bond between the 3'‐OH of the RNA strand tail and the 5'‐monophosphate of the RNA strand head in an ATP‐dependent manner. For a chemically synthesized linear RNA precursor, the 5'‐monophosphate can be incorporated during the synthesis or added subsequently using T4 polynucleotide kinase. However, linear RNA precursors produced via IVT typically begin with a 5'‐pppG moiety due to inherent transcriptional features. Consequently, prior to ligation reaction, the 5'‐terminus requires dephosphorylation using phosphatases and subsequent monophosphorylation [41]. Alternatively, GMP‐primed IVT provides a viable approach for directly obtaining 5'‐monophosphorylated linear RNA precursors that can be readily utilized for circularization [42].

T4 DNA ligase exhibits the ability to join nicks in double‐stranded duplexes, including DNA/DNA and DNA/RNA hybrids. To ensure efficient ligation, it is crucial to have a complementary DNA bridge with a minimum of ten nucleotides on each side of the ligation junction [41]. Furthermore, T4 DNA ligase demonstrates limited tolerance for mismatches at the ligation junction. Therefore, incorporating a perfectly complementary DNA splint for the RNA sequence can significantly enhance ligation efficiency [43]. Despite these optimizations, the turnover efficiency of T4 DNA ligase on RNA substrates remains low, making it less suitable for circRNA preparation.

T4 RNA ligase 1 is commonly utilized for RNA ligation due to its direct catalytic ability in joining the 3'‐OH of an RNA strand tail with the 5'‐monophosphate of an RNA strand head. The addition of an imperfect complementary RNA splint into the reaction system facilitates circRNA synthesis [34, 37]. This approach reduces the distance between 3'‐OH and 5'‐monophosphate, while also preventing unfavorable folding patterns at the ligation junction that could hinder the ligation reaction. To achieve efficient circRNA synthesis without a splint, an optimized design ensuring an appropriate secondary structure of the linear RNA at reaction ends can expedite the ssRNA ligation [44]. By employing this method, Carmona et al. successfully accomplished efficient RNA circularization up to 4 kb in size [44]. Additionally, the choice of terminal nucleotides for ligation also influences the efficiency, with cytidine and adenosine exhibiting preferences at the 5' and 3' ends, respectively [45].

The activity of T4 RNA ligase 2 in joining RNA nicks with a double‐stranded structure is higher compared to the ends of ssRNA, as reported by previous studies [46]. This distinctive property has been effectively harnessed in vitro to circularize RNA substrates, which are held together through the utilization of DNA/RNA splint or facilitated by meticulously designed internal secondary structures at the reaction ends [40–42, 47]. Nevertheless, irrespective of whether the reaction is catalyzed by T4 RNA ligase 1 or T4 RNA ligase 2, the ligation process dependent on secondary structure will be influenced by distinct RNA sequences. Moreover, all these enzymatic ligation strategies are susceptible to significant intermolecular end‐joining side reactions, thereby complicating the acquisition of pure circRNAs [41]. To minimize intermolecular ligation, it is generally preferable to maintain a linear precursor RNA in a relatively large reaction volume, thereby enhancing the yield of desired circRNA products and mitigating unwanted side reactions [40, 43, 47].

### 3.3 Ribozyme Technology

Ribozymes are a class of RNAs that possess the remarkable ability to catalyze specific biochemical reactions. The discovery of ribozymes challenges the conventional notion of protein‐based enzymes, as they demonstrate that RNA can also exhibit inherent catalytic properties. These catalytic RNA molecules have been identified in diverse organisms, including viruses, bacteria, archaea, and eukaryotes [34]. Based on their size and catalytic mechanisms, ribozymes can be classified into two distinct categories: large ribozymes, such as RNase P, group I introns, and group II introns; and small ribozymes, including hairpin ribozymes (HPRs), HDV ribozymes, hammerhead ribozymes (HHRs), *Neurospora* Varkud satellite (VS) ribozymes, glucosamine‐6‐phosphate synthase (glmS) ribozymes, among others [48]. Among these ribozymes, the group I intron, group II intron, and hairpin ribozyme have been utilized as engineering tools to facilitate RNA circularization on a preparative scale in vitro [20, 49]. Compared to chemical and enzymatic ligation methods, the utilization of ribozyme technology offers several advantages, including enhanced circularization efficiency for larger linear RNA precursors, simplified reaction conditions, and purification methods. Incorporating ribozymes into circulation procedures can thus yield improved outcomes with increased efficiency and ease of implementation.

#### 3.3.1 Hairpin Ribozyme

**FIGURE 3:**
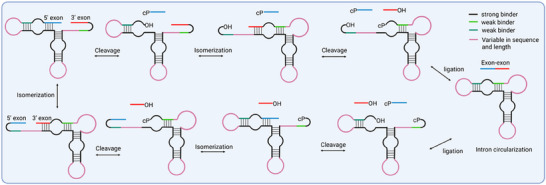
HPR‐mediated self‐splicing scheme. The HPR can adopt two alternative conformations in its initial state, and different desired sequences can be inserted into variable regions. In the final splice products, the 5' exon and 3' exon are ligated, resulting in cyclization of the HPR. Each reaction step in HPR‐mediated self‐splicing is reversible.

As previously mentioned, the rolling‐circle mechanism employed by circRNA genomes of subviral agents has served as an inspiration for in vitro circRNA synthesis. In this model, HPRs that catalyze RNA processing reactions are integrated into specific circRNA genomes. Upon transcription, the local RNA sequence containing HPR can adopt two alternative active conformations, resulting in excision of the 5' and 3'‐ends and generation of multiple 5'‐OH and 2',3'‐cyclic phosphate (cP) intermediates from the precursor RNA concatemer. These intermediates are subsequently circularized through an HPR‐mediated self‐ligation reaction (Figure 3) [45, 50].

In 1998, Kool and Diegelman proposed a technique for generating circRNAs through an in vitro rolling‐circle reaction, utilizing the intrinsic cleavage and ligation activities of the HPR [51]. This approach involves employing RNA polymerase to perform rolling circle transcription from a circular single‐stranded DNA template, resulting in a linear RNA concatemer containing the HPR element. Subsequently, the linear RNA undergoes spontaneous cleavage and ligation processes, leading to the production of circRNAs [51]. Moreover, comprehensive investigations have been undertaken to investigate the influence of diverse factors, including temperature, ions, and supplementary cofactors, in order to enhance HPR activity [52].

Currently, HPR strategies are primarily utilized for the preparation of small circRNAs, although there is potential for expanding its application to larger circRNAs by manipulating variable regions [45, 47]. In comparison to other RNA circularization systems, the HRP system does possess certain limitations that need to be addressed in future applications. Notably, the sequence of the HPR ribozyme remains embedded within the final generated circRNAs, resulting in the retention of active ribozymatic activity in these circRNAs [45]. Consequently, a dynamic equilibrium between cleaved and ligated conformations is maintained in these circRNAs.

#### 3.3.2 Group I Intron

**FIGURE 4:**
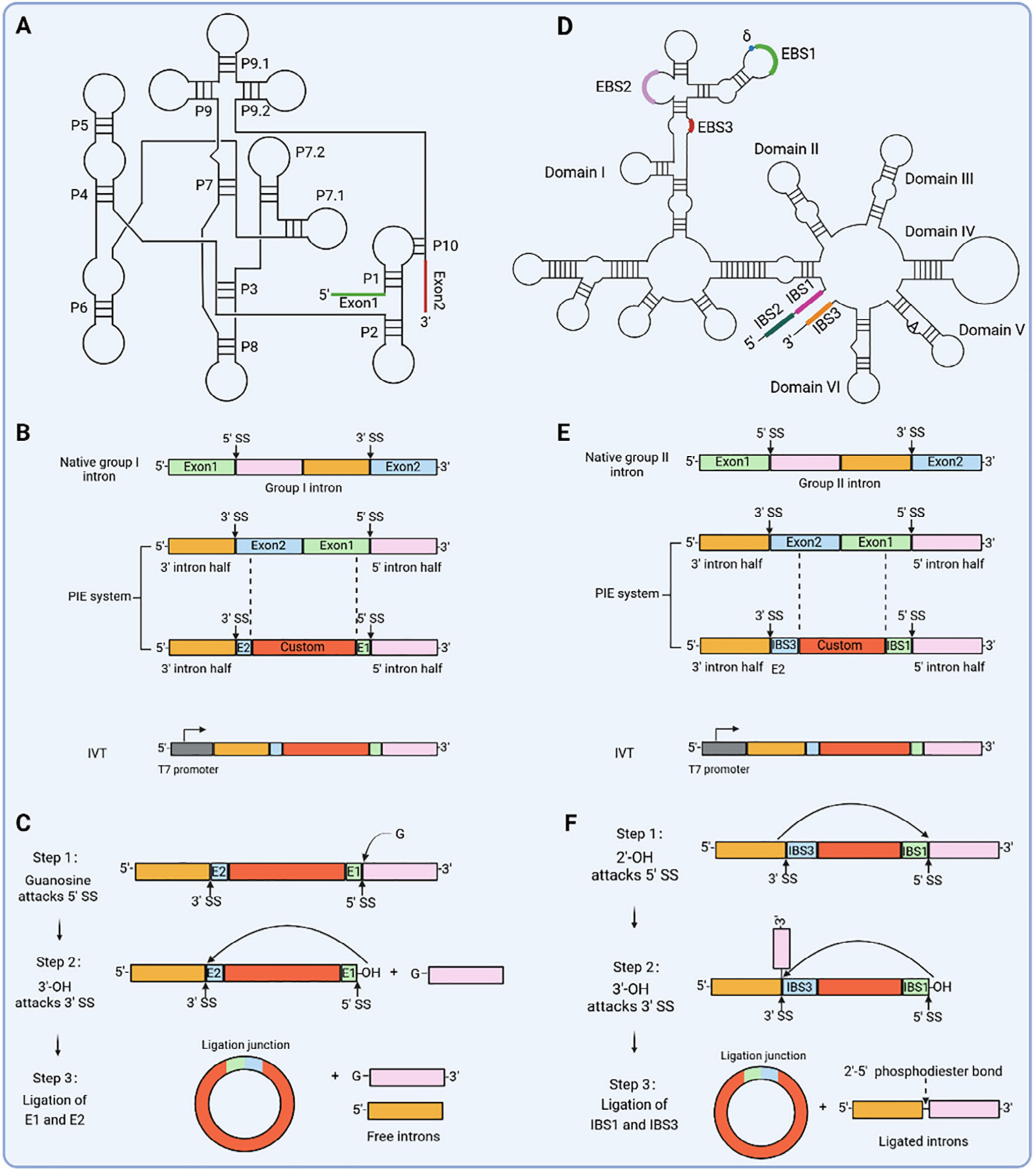
Ribozyme technology applied for RNA circularization. (A) The consensus secondary structure of group I introns, including conserved (P1–P10) base pairing, is sequentially numbered and indicated. Exon sequences are visually highlighted. (B) A schematic diagram illustrating the engineered group I PIE system is presented, wherein custom sequences are strategically incorporated between E1 and E2, while the T7 promoter located at the 5'‐end responds to RNA polymerase, thereby generating linear precursor RNA. (C) Proposed splicing pathway of group I PIE based on the native splicing mechanism is presented. (D) The secondary structure of group II introns consists of six domains (I–VI) arranged around a central wheel, with the exon sequences represented by bold lines. Conserved tertiary base pairing interactions are observed between IBS1‐EBS1, IBS2‐EBS2, and IBS3‐EBS3/δ. Additionally, domain VI contains the conserved adenosine residue responsible for initiating the splicing reaction. (E) The engineered group II PIE system is illustrated in a schematic diagram, where custom sequences are inserted between IBS1 and IBS3. The T7 promoter is utilized for IVT. (F) A proposed splicing pathway of group II PIE is presented based on the native splicing mechanism.

Group I intron ribozymes have been identified in various organisms, including viruses, bacteria, and certain eukaryotes, particularly within mitochondrial, chloroplast, and rRNA genes [53]. These ribozymes exhibit self‐splicing activity that relies on GTP and Mg^2+^ as cofactors [20]. The consensus secondary structure of the group I introns comprises ten conserved paired regions denoted as P1–P10 (Figure 4A) [54]. The intron internal guide sequence (IGS) recognizes the 5' exon sequence via a 4∼6 nt base pairing, thereby forming the P1 region that initiates the two‐step splicing mechanism for group I intron excision from pre‐RNA [55]. The GTP cofactor binds to the G‐binding site in the P7 helix and functions as a nucleophile, initiating an attack on the 5' splice site [56]. This attack leads to cleavage of the phosphate bond that connects the intron with the 5' exon. Subsequently, the released 3'‐OH group of the 5' exon undergoes an attack on the 3' splice site located in the P10 region, which is formed through base pairing between IGS and the sequence of the 3' exon, resulting in exon ligation and intron release [55].

Based on this mechanism, researchers have developed a modified self‐splicing system called PIE for the in vitro preparation of circRNAs (Figure 4B,C) [20, 57]. In 1992, Puttaraju and Been demonstrated the adaptability of group I intron system derived from *Anabaena* PCC7120 pre‐tRNA^Leu^ and *Tetrahymena* pre‐rRNA in generating circRNAs from linear intron‐containing precursors [58]. Following that, in 1994, Ford and Ares employed a permuted intron derived from the thymidylate synthase (*td*) gene of T4 bacteriophage to achieve RNA circularization, marking a significant milestone in this field [59]. As a result, permuted T4 *td* and *Anabaena* pre‐tRNA^Leu^ genes have become widely adopted as essential frameworks for circularizing diverse exonic sequences. By utilizing the PIE system derived from *Anabaena* pre‐tRNA^Leu^, researchers have successfully generated circRNAs up to 5 kb in length [20], surpassing the circularization capacity achieved by chemical and enzymatic ligation strategies. However, it is essential to note that the employment of the group I PIE system typically leads to the introduction of a scar sequence, which consists of a small number of nucleotides from the 5' exon (E1) and 3' exon (E2), into the final circRNA products [20].

#### 3.3.3 Group II Intron

Group II introns, mobile genetic elements mainly found in bacterial and organellar genomes, are considered ancestors of retrotransposons and spliceosomal introns in eukaryotes [60]. These typical group II introns exhibit six secondary structural domains (domain I–VI) with highly conserved sequence and share an architecturally conserved tertiary structure (Figure 4D) [61]. The majority of group II introns in bacteria, as well as approximately half in mitochondria and chloroplasts, encode an intron‐encoded protein (IEP) located within the loop of domain IV. This IEP plays a crucial role in splicing by stabilizing the structure of catalytically active intron RNA [60]. In vitro experiments have demonstrated that self‐splicing of group II introns can occur with correct folding of intronic RNA structure and Mg^2+^ [62], albeit at a slow rate and under non‐physiological conditions involving high concentrations of monovalent salt and/or Mg^2+^ [49]. These experimental conditions suggest that IEP is indispensable for facilitating splicing activity in group II intron RNA. Moreover, a multitude of organellar group II introns display structural anomalies that hinder the activity of ribozymes, thereby restricting their utility in RNA circularization [63].

Group II intron‐mediated self‐splicing involves two transesterification reactions to excise themselves from pre‐RNA. In the initial step, a bulged adenosine residue in domain VI acts as a nucleophile, utilizing its 2'‐OH to attack the 5' splice site and form a lariat structure. Subsequently, the free 3'‐OH of the 5' exon initiates an attack on the 3' splice site, resulting in exon ligation and removal of the lariat RNA [64]. Additionally, a water molecule can also serve as a nucleophile, initiating the initial transesterification reaction by attacking the 5' splice site [62]. A group II intron self‐splicing system designed in a PIE manner exhibits efficient RNA circularization in vitro (Figure 4E‐F). Similarly to group I PIE system, it still retains a scar sequence derived from the 5' exon (IBS1) and 3' exon (IBS3) in the final circRNA products [49].

## 4 Translational Mechanism of circRNAs

In eukaryotes, pre‐mRNAs undergo essential modifications, including 5' capping, 3' polyadenylation, and intron splicing, to form mature and functional mRNA molecules. The process of 5' capping is crucial for the translation of mRNAs to proteins by ribosomes. However, covalently closed circRNAs lack this modification, necessitating the utilization of cap‐independent mechanisms for their translation initiation primarily. Despite the identification of numerous circRNAs in eukaryotic cells, there remains a lack of understanding regarding the extent and functions of circRNA translation. Currently, four mechanisms have been confirmed for circRNA translation: (a) rolling circle translation; (b) N^6^‐methyladenosine (m^6^A)‐mediated translation; (c) internal ribosome entry site (IRES)‐driven translation; (d) Artificially designed cap‐dependent translation.

### 4.1 Rolling Circle Translation

**FIGURE 5:**
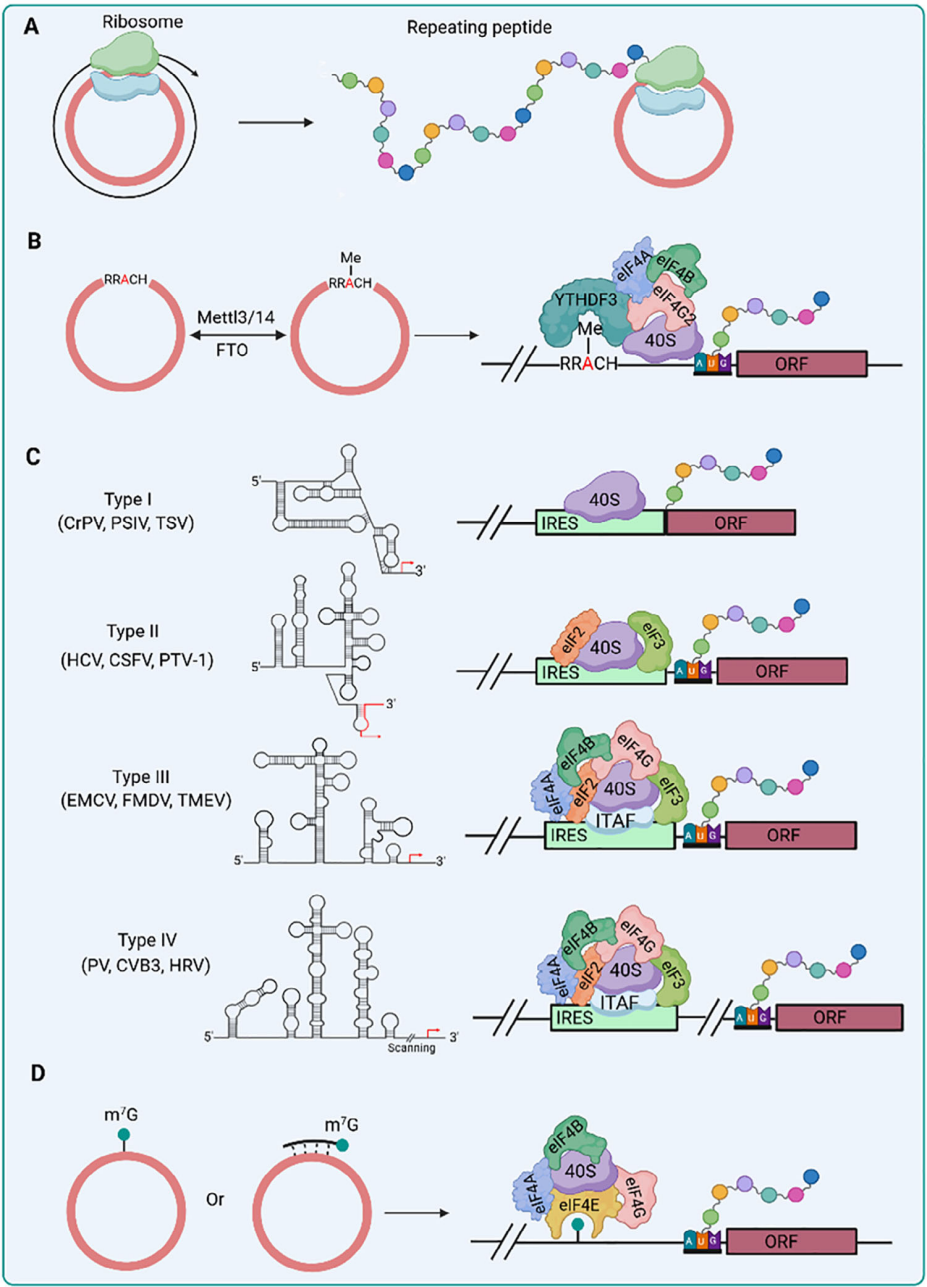
CircRNAs undergo translation through the cap‐dependent/independent pathway. (A) Rolling circle translation of a repeating peptide on a circRNA template. (B) The process of m^6^A modification in circRNAs involves the METTL3/METTL14 complex for addition and FTO for removal of the m^6^A mark. YTHDF3 facilitates m^6^A recognition, recruiting elF4G2 to m^6^A site, thereby initiating translation. (C) Four types of IRES‐mediated translation have been identified. Type I and II IRESs directly interact with 40S ribosome, while Type III and IV IRESs indirectly interact with 40S ribosome through the assistance from eIFs and ITAFs. Several representative viral IRESs are listed as examples. (D) Cap‐dependent translation of circRNA is achieved by introducing an m^7^G cap through covalent or non‐covalent attachment.

In 1998, Perriman and Ares demonstrated the translatability of circRNA molecules in vivo in *Escherichia coli*, pioneering a significant advancement in the field. Their findings revealed that circRNA exhibited lower translation efficiency compared to linear RNA due to less efficient translation initiation [65]. Considering that translation initiation is the rate‐limiting step in protein expression, Abe et al. devised a novel circRNA translation system devoid of stop codons by mimicking the rolling circle amplification (RCA) process observed during DNA replication (Figure 5A). Consequently, this system effectively achieves translation by significantly reducing the frequency of re‐initiation processes in both prokaryotic and eukaryotic systems [66]. Recent studies have revealed that approximately 50% of translatable endogenous circRNAs undergo rolling circle translation, as evidenced by further systematic assays demonstrating a significant enrichment of IRES‐like short elements in these endogenous circRNAs [67]. IRES‐like elements can be recognized by trans‐acting factors to facilitate the translation of circRNAs [67], thereby providing novel insights into both natural and artificial circRNA translation regulation. However, concerns arise regarding the rolling circle translation method employed for circRNA synthesis. First, this approach may result in proteins containing concatemeric repeats, potentially compromising their folding and stability. Additionally, the presence of diverse alternative ORFs within circRNAs could be translated independently through IRES‐like elements, resulting in a complex protein mixture.

### 4.2 m^6^A‐Mediated Translation Initiation

The m^6^A modification is the predominant internal mRNA modification observed in eukaryotes [68], widely present within the RRACH (R = G or A; H = A, C, or U) consensus motif [69]. This dynamic modification process involves writer proteins primarily represented by the METTL3/METTL14 complex and eraser proteins such as FTO and ALKBH5 [70].

A study conducted by Yang *et al.* revealed that he translation of circRNA driven by m^6^A is cell type‐independent, and a single m^6^A site is sufficient to initiate translation [71]. YTH domain family protein 3 (YTHDF3) has been identified as a crucial factor in recognizing m^6^A and recruiting eIF4G2 to the m^6^A site, thereby initiating translation (Figure 5B) [71, 72]. Furthermore, incorporation of artificial m^6^A modifications in circRNAs has been shown to enhance their resistance to nucleases, indicating that this modification confers improved stability and sustained translation of circRNAs [73]. These findings underscore the critical role of m^6^A modification in regulating circRNA translation and stability, with potential implications for various cellular processes and immune responses [74]. Notably, the initiation of m^6^A‐dependent translation appears to be finely regulated under certain stress conditions such as heat shock [71a]. However, the mechanism underlying m^6^A‐initiated translation remains poorly understood, presenting challenges such as determining the optimal initial position for translation and addressing potential frameshift mutations.

### 4.3 IRES‐Driven Translation Initiation

The IRES is a cis‐acting RNA motif originally discovered in the *Picornaviridae* family viruses, such as encephalomyocarditis virus (EMCV) and poliovirus (PV) [75], initiating translation via a cap‐independent pathway. Despite lacking a 5'‐cap structure like mRNA, IRES is capable of directly interaction with ribosomes or indirectly interaction with ribosomes through IRES‐transacting factors (ITAFs) in order to facilitate protein synthesis in conjunction with canonical initiation factors. In recent years, extensive efforts have been devoted by researchers to identify and characterize the IRES elements within viral genomes and cellular mRNA [76], thus highlighting the significance of cap‐independent RNA translation as an essential aspect of protein synthesis. In eukaryotes, the initiation of protein translation via the cap‐dependent mechanism necessitates a substantial amount of energy due to the involvement of numerous initiation factors and multiple steps [77]. Consequently, in response to unfavorable cellular stress conditions, there is a universal cessation of the standard protein synthesis process to ensure cell survival [78]. As a consequence, the cap‐independent translation driven by IRES is deemed to be an alternative mechanism for protein synthesis under stress conditions when cap‐dependent protein translation is inhibited [79]. The insertion of IRES elements derived from viruses such as coxsackievirus B3 (CVB3) and echovirus 29 (EV29) into artificially designed circRNAs has emerged as the preferred strategy to enable cap‐independent initiation in eukaryotes [22, 73]. However, the detrimental effects of these viral sequences on host cells have received limited attention thus far. In this regard, employing humanized IRES with robust and stable translation capabilities presents a superior approach that circumvents the import of viral nucleotide sequences. Notably, in certain instances, IRES engineering has been implemented to enhance their translational efficiency by strategically incorporating Apt‐eIF4G, an aptamer that recruits eIF4G and thereby improves translation when inserted within the designated IRES region [73, 80].

Based on their structural characteristics and translation initiation mechanisms, viral IRESs can be classified into four types [76, 79]. Among these types, type I IRESs (e.g., Cricket paralysis virus, CrPV; Plautia stali intestine virus, PSIV; Taura syndrome virus, TSV) possess a unique feature as they are capable of directly interacting with the 40S ribosome without requiring additional cofactors. Notably, they demonstrate an exceptional ability to initiate translation independently of any proteins, even from non‐AUG start codons [79a]. However, translation initiation at non‐AUG positions may result in undesired frameshift mutations. Type I IRESs are typically characterized by a compact structure comprising three essential pseudoknots (PKI, PKII, and III) and two conserved stem loops [81]. It is noteworthy that the structure of the IRES‐ribosome complex reveals that PKI mimics a codon‐anticodon interaction, facilitating its independent loading into the A site of the ribosome without relying on initiator Met‐tRNAi [82]. On the contrary, type II IRESs (e.g., Hepatitis C virus, HCV; Classical swine fever virus, CSFV; Porcine teschovirus 1, PTV‐1) are capable of interacting with the 40S subunit; however, their translation initiation activities predominantly depend on canonical eukaryotic initiation factors (eIFs), including eIF2, eIF3, and Met‐tRNAi, to ensure efficient translation initiation [79, 83].

In contrast to type I and type II IRESs, both type III (e.g., EMCV; Foot‐and‐mouth disease virus, FMDV; Theiler's mouse encephalomyelitis virus, TMEV) and IV IRESs (e.g., Poliovirus, PV; CVB3; Human rhinovirus, HRV) do not directly interact with 40S ribosome. Instead, they depend on specific canonical initiation factors and ITAFs to recruit the 40S ribosome for translational initiation [79a]. The key distinction between them lies in their translational initiation mechanisms. Type IV IRESs initiate translation at the AUG codon through a 40S ribosome scanning process within the 5' untranslated region. In contrast, type III IRESs do not require this scanning process (Figure 5C) [84]. Notably, due to the ribosome scanning of Type IV IRESs, there exists an opportunity to introduce additional nucleotides, such as a Kozak sequence between the IRES and AUG codon to enhance protein expression. Hence, Type IV IRESs are extensively employed for the design of protein‐coding circRNAs. Despite significant advancements, the intricacies of IRES‐mediated translation continue to be a subject of ongoing investigation, necessitating further research to comprehensively elucidate its underlying mechanisms [73].

### 4.4 Artificially Designed Cap‐Dependent Translation

In eukaryotes, the 5' end of linear mRNA possesses an m^7^G cap structure and utilizes its interaction with eIFs, among which the direct cap‐binding eIF4E protein is included, for translation. Considering that the translation initiation efficiency mediated by IRES is lower than that of cap‐dependent translation initiation [85], in an effort to enhance the protein translation ability of circRNA, Chen et al. introduced a modified cap into circRNA lacking IRES, achieving efficient translation of circRNA through eIF4E‐dependent mechanisms [86]. Although the unnatural triazole linkage between the branched cap and the circRNA substitutes the phosphodiester bonds, this seemingly does not affect the translation of circRNA. These findings provide additional insights into current translation initiation models. Fukuchi et al. recently not only replicated the research results of Chen et al. but also accomplished the translation of circRNA through the non‐covalent attachment of an m^7^G cap to the circRNA by hybridization with an m^7^G cap‐containing oligonucleotide. Further studies have demonstrated that circRNA synthesized in vitro can be translated with the assistance of intracellular mRNA or long non‐coding RNA through interaction after transfection, allowing circRNA to act as a reporter for detecting the presence of these RNA molecules in cells and significantly expanding the application scope of circRNA (Figure 5D) [87].

## 5 Engineered Platforms for circRNA Preparation

Strategies for in vitro circRNA synthesis have been developed and applied for analytical scientific studies, the large‐scale preparation of circRNA still remains a bottleneck due to its high cost for industrial applications [88]. In order to address this challenge, several engineered platforms have been devised with the aim of minimizing costs by optimizing circRNA technologies.

### 5.1 ORNA System

**FIGURE 6:**
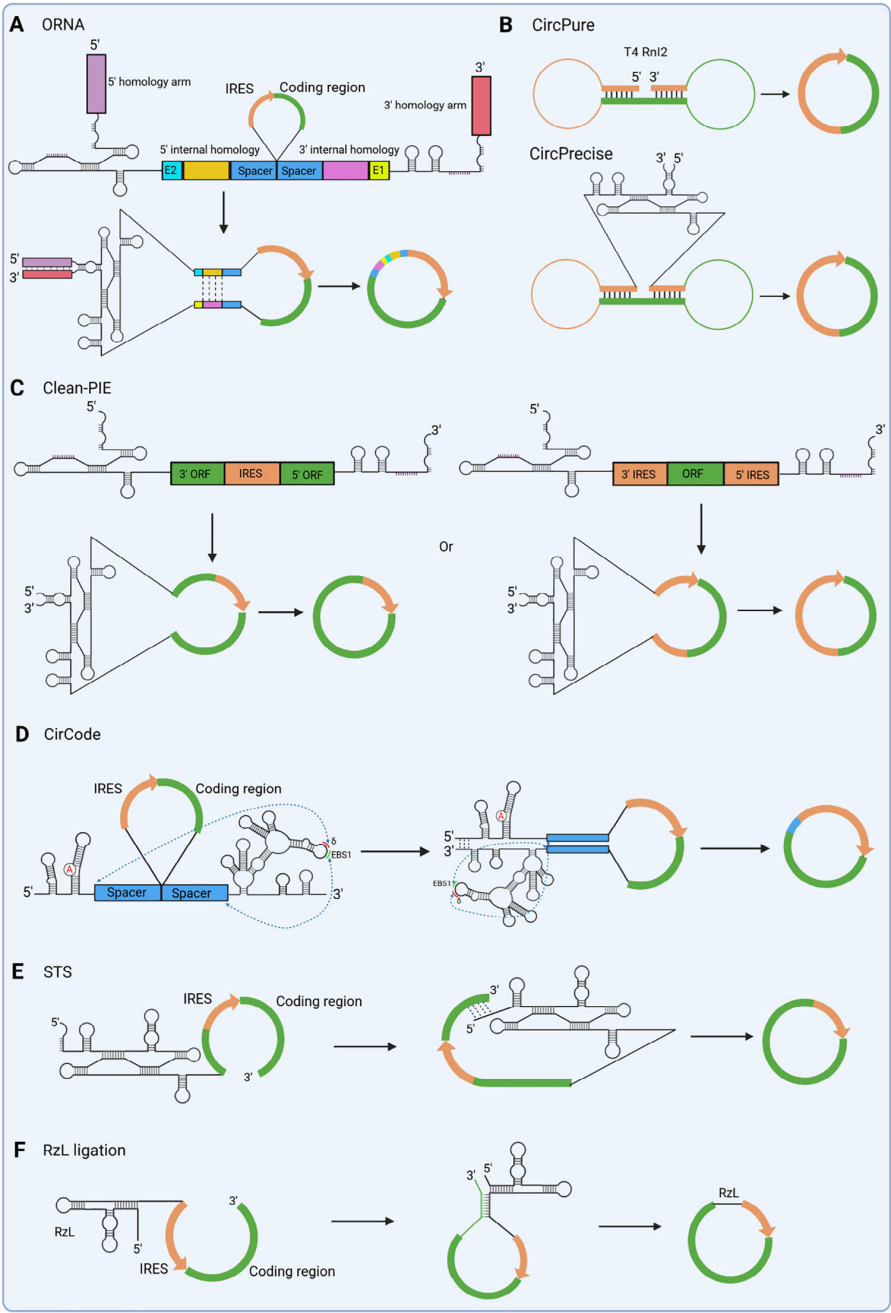
Engineered precursor RNA design and circularization process (A) ORNA: The group I intron is divided into two halves and systematically assembled with IRES, coding region, external and internal homology arms, E1 and E2 motifs, as well as spacers to generate a precursor RNA for subsequent circularization. Homology arms play a crucial role in enhancing the efficiency of linear RNA circularization, while spacers facilitate the translation of circRNAs. (B) CirPure and CirPrecise system: Utilizing AI technology to predict the secondary structure of selected circRNA and guide the design of precursor RNA for intramolecular base pairs formation, preferably employed by T4 RNA ligase 2 or PIE system. (C) Clean‐PIE: A customized sequence comprising both IRES and coding region is designed using a scoring system to make the splice site of the group I PIE system within either the coding region or IRES sequence. (D) CirCode: The self‐splicing group II intron of ctSLP is divided into two fragments, with a customized sequence comprising an IRES and the coding region of a target gene seamlessly inserted between the split introns. To achieve scarless ligation, modifications are introduced to optimize the IBS1‐EBS1 and δ‐EBS3 pairs. Furthermore, spacers are thoughtfully incorporated to enhance circularization efficiency and translation activities. (E) STS: A linear RNA precursor is generated wherein a customized sequence follows the intron ribozyme; importantly, at its 3' end, this customized sequence can form an intact P1 helix through pairing with the IGS of the intron ribozyme during the RNA circularization process. (F) RzL ligation: A cis‐acting ligase ribozyme (RzL) is used to generate circRNA through direct ligation of 5'‐ppp and 3'‐OH.

In 2018, Aderson et al. pioneered the development of an ORNA system utilizing Anabaena group I PIE for efficient RNA circularization, thereby enabling the production and translation of circRNAs up to 5 kb in length (Figure 6A) [20]. During the ASGCT (American Society of Gene & Cell Therapy) conference in 2022, they presented their groundbreaking achievement of successfully generating circRNA exceeding 10 kb using the ORNA system [89]. Their optimized ORNA system involved the simultaneous incorporation of external and internal homology arms at engineered 3' and 5' intron halves, resulting in a significantly augmented circularization efficiency. Moreover, to ensure stable circRNA expression across diverse cell types, a ploy(A) or ploy(AC) spacer was introduced into the final circRNA products [20].

The incorporation of a poly(A) or poly(AC) sequence holds the potential to enhance translation initiation efficiency by potentially facilitating the recruitment of polyadenylate binding proteins, like their role in linear mRNA translation [90]. Additionally, the unstructured spacer effectively segregates the IRES from other elements, such as paired internal homology arms and exon–exon splice junctions, thereby minimizing steric hindrance that may impede eIF binding to IRES [20]. However, it is imperative to consider that the utilization of this system may inevitably lead to the presence of additional nucleotide sequences in the circRNA products, which could potentially raise concerns regarding their immunogenicity upon delivery into cells. Thorough assessment and further investigations are warranted to evaluate and address this aspect for ensuring safe and efficacious therapeutic applications.

### 5.2 CircPure Ligation

In 2020, Liang et al. introduced a preparative‐scale method, later named CircPure by Geneseed company, for the synthesis circRNAs utilizing T4 RNA ligase 2 (Figure 6B) [47b]. This approach commences with the utilization of artificial intelligence (AI) technology to predict the secondary structure of the target circRNA, encompassing both the IRES and encoding region. Consequently, a strategically designed precursor RNA was employed to facilitate preferential intramolecular base pairing that aligns precisely with the catalytic kinetics of T4 RNA ligase 2. As a result of this innovative approach, the authors successfully achieved exceptional circRNA yields (>90%) while minimizing the formation of polymeric by‐products.

Following this successful approach, the researchers further expanded their methodology to encompass the group I PIE system, known as CircPrecise, which effectively facilitates circRNA preparation (Figure 6B) [47b]. Notably, circRNAs generated through both the CircPure and CircPrecise methods exhibit precise composition devoid of any additional residues. However, limited information is currently available regarding their expression patterns and immunogenicity resulting from intramolecular base pairing. Furthermore, it is imperative to acknowledge that this system may manifest variations attributable to the diverse structural characteristics of targeted sequences, which could potentially impact overall efficacy and outcomes. Further investigation is warranted to gain a more comprehensive understanding of the expression profiles and immunogenic properties of circRNAs generated through these innovative methodologies.

### 5.3 Clean‐PIE Strategy

In order to address concerns regarding potential immunogenicity and optimize efficient protein translation from circRNA molecules, Qiu et al. developed a novel and sophisticated approach known as the clean‐PIE strategy. This innovative method involves concealing the splice site of T4 *td* PIE within either an ORF or IRES sequence using a scoring system, resulting in the generation of a circRNA molecule devoid of any other intron and exon sequences (Figure 6C) [91].

Interestingly, circRNAs derived from concealing the splice site within the ORF exhibited a propensity for reduced protein expression. However, this concern was effectively addressed by incorporating a moderate poly(AC) motif into the circRNAs, resulting in significantly enhanced levels of protein expression. In contrast, circRNAs generated by concealing the splice site within the IRES demonstrated robust protein expression capability even without any additional sequences [91]. While these approaches reduce potential side effects resulting from extra nucleotides, they impose more stringent requirements for accurately analyzing RNA secondary structures and designing precursor RNAs. Even so, these findings underscore the significance of employing the Clean‐PIE strategy to optimize circRNA‐based therapeutics and present a promising avenue for future research in molecular biology and biotechnology.

### 5.4 CirCode Technology

In 2022, Chen et al. introduced an innovative platform named CirCode, which utilizes a group II intron derived from the surface layer protein of *Clostridium tetani* (ctSLP) (Figure 6D) [49]. The research team meticulously engineered and synthesized the PIE sequence by strategically splitting the intron at the loop region of D4 following extensive experimentation.

The CirCode platform incorporates a customized exon flanked by two half‐introns split at the D4 loop region. This precise arrangement ensures the formation of a well‐paired structure, thereby maintaining proper folding and activity of the intron. Furthermore, to minimize potential interference from the scar sequence (IBS1 and IBS3), modifications were made to the PIE system. These modifications involve establishing base pairs between EBS1 and the upstream sequence of EBS1 (*δ*) with the 3' end of the customized sequence and 5' end of IRES, respectively [49, 62].

These design optimizations facilitated the generation of scarless circRNAs, albeit with an initially lower circularization efficiency compared to group I PIE. To enhance circularization efficiency, the researchers introduced spacers containing IRES‐like short elements [49, 67]. This strategic addition resulted in a remarkable increase in circularization efficiency, reaching up to 83%, and significantly improved the translation activities of the generated circRNAs. The CirCode platform represents a pioneering advancement in the field, showcasing its potential for shaping future research in circRNA manipulation and therapeutic applications.

### 5.5 STS Reaction

In a recent study, Lee et al. developed a novel in vitro circRNA engineering method through end‐to‐end self‐targeting and splicing (STS) reaction employing *Tetrahymena* group I intron ribozyme (Figure 6E) [92]. In their approach, an optimal sequence within the gene of interest (GOI) was meticulously selected as the target site to pair with the IGS of the intron ribozyme, facilitating the formation of an intact P1 helix. Importantly, this STS reaction no longer relies on the presence of the P10 helix. Furthermore, this end‐to‐end STS reaction avoids introducing any extraneous fragments, such as intronic scars that may be generated by other systems, like ORNA, into the final circRNA products. These scar sequences are considered potential triggers for undesired innate immune responses in cells, a finding that has been confirmed [93]. The RNA circularization efficiency of the STS reaction was found to be comparable to that of the PIE reaction method [92].

Qi et al. designed a cis‐splicing system similar to STS and also verified this point [94]. Notably, these systems obviate the need for additional spacer elements that are crucial for ensuring the correct ribozyme structure in the PIE system. This might mainly be ascribed to an intact ribozyme core situated at one end of the precursor, rather than relying on the correctly reconstructed ribozyme structure with the aid of homology arms and spacer elements.

### 5.6 RzL‐Mediated Ligation

In the aforesaid ribozyme‐mediated RNA circularization process, the splicing activity of the ribozyme is mainly utilized to obtain circRNA. In contrast, Su et al. developed a novel circRNA synthesis approach by exploiting the ligase activity of the ribozyme [95]. In this strategy, an RNA precursor containing a cis‐acting ligase ribozyme (RzL) and substrate sequence was transcribed. The RzL enables the 5'‐triphosphate (ppp)‐bearing RzL to directly and autonomously join the 3'‐end corresponding substrate sequence during IVT (Figure 6F). Evidently, the RzL strategy highly depends on the ribozyme‐substrate RNA pairing to generate circRNA, and simultaneously, there will be no byproduct RNA in the product. This is similar to RNA circularization mediated by T4 RNA ligase, but it is not feasible in the circRNA synthesis process mediated by the PIE system. Nevertheless, RzL‐mediated RNA ligation will undoubtedly incorporate RzL itself into the final synthesized circRNA, which might raise concerns about the immunogenicity of circRNA obtained by this method, and this requires detailed research in future studies.

## 6 Quality Control and Characterization of circRNAs

Regardless of the method employed for circRNAs production, such as permuted autocatalytic introns or enzyme‐mediated ligation, the final reaction products often exhibit a variety of impurities. This can potentially result in unsatisfactory outcomes or conflicting conclusions regarding the immunogenicity of synthetic circRNAs upon cellular delivery [21, 74, 93], possibly attributable to suboptimal quality of circRNA products [49, 96]. In addition, the intricate composition of custom sequences may occasionally give rise to unexpected splicing reactions. To ensure the integrity and suitability of circRNAs for cellular applications, a variety of specialized techniques have been developed to facilitate comprehensive quality control and characterization.

### 6.1 CircRNA Analysis and Identification Methodologies

**FIGURE 7:**
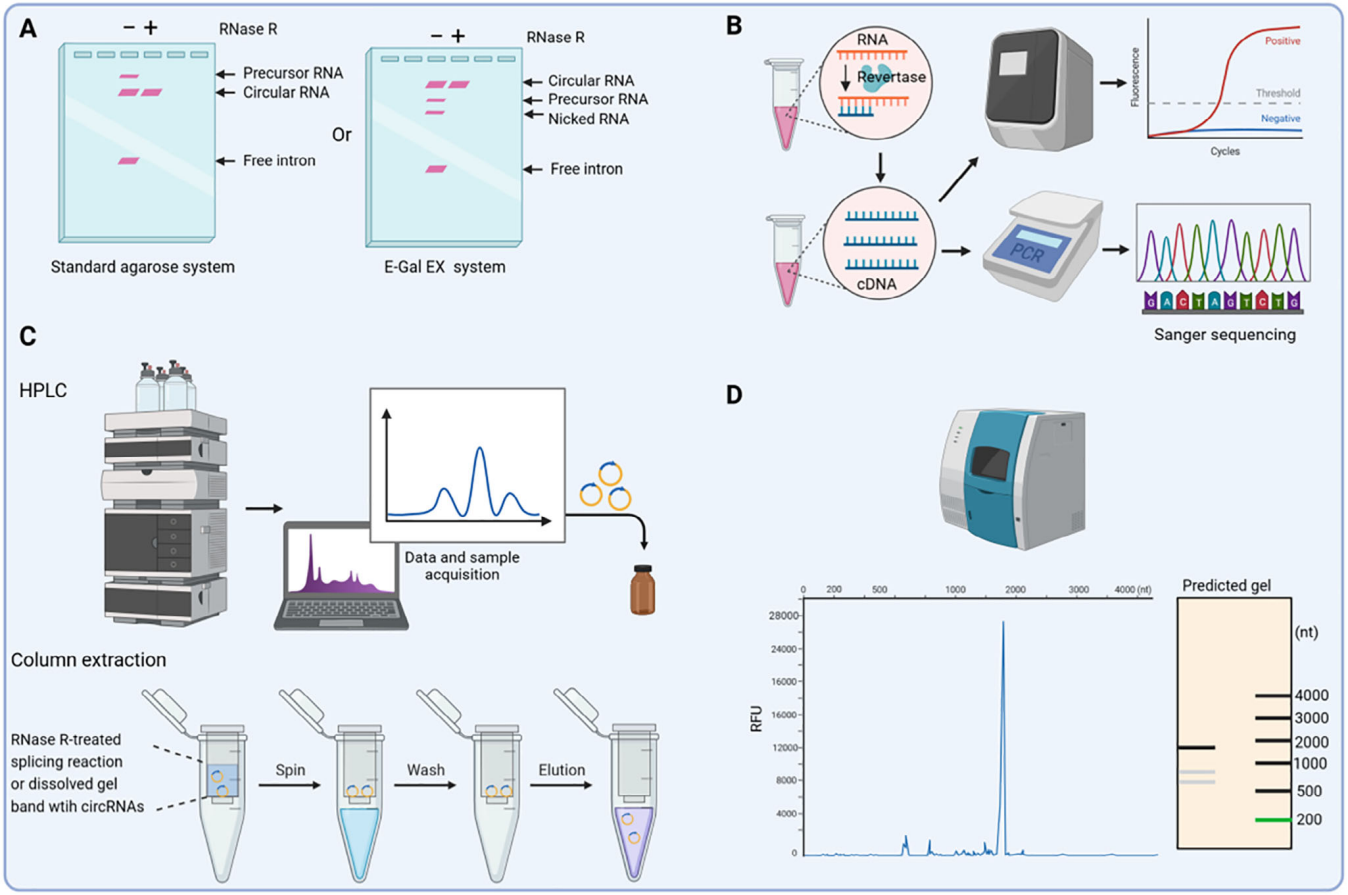
Multiple technologies are developed to analyze and purify circRNAs from the reaction mixtures. (A) Gel electrophoresis analysis of the entire reaction products with or without RNase R treatment. (B) RT‐qPCR is used to validate the existence of circRNA, while Sanger sequencing is utilized to confirm the occurrence of ligation at the intended position. (C) For circRNA purification, either column extraction or HPLC method was employed. (D) The purity of circRNA was assessed using capillary electrophoresis.

Polyacrylamide and agarose gel electrophoresis are commonly employed techniques for the analysis of circRNA reaction mixtures. High‐resolution separation can be achieved through polyacrylamide gel electrophoresis with a high concentration of urea (Urea‐PAGE), allowing for the discrimination of base‐level differences [44, 58]. However, this method may not be suitable for large circRNA analysis. In recent studies, agarose gel electrophoresis has gained popularity due to its effective capability in separating RNA across a wide range. While self‐cast standard agarose gels can separate the final products from ribozyme‐catalyzed splicing reactions, they may present challenges in distinguishing nicked RNA bands from circRNA bands of identical molecular weight [97]. To address this issue, E‐Gel EX agarose gel electrophoresis was developed by Aderson et al. [20], enabling precise discrimination between circular and nicked RNA components during circularization reactions. However, it is important to note that the interpretation of migration patterns under specific conditions requires careful consideration (Figure 7A) [97].

RNase digestion assays are routinely employed to identify the circular products in the splicing reaction. CircRNAs exhibit higher resistance to RNase R digestion compared to linear RNA, enabling their retention in RNase R‐treated splicing reactions [73]. Further digestion of the RNase R‐treated splicing reaction with oligonucleotide‐targeted RNase H generates a distinct single band, distinguishing it from the two bands produced by RNase H‐digested linear precursor [20].

In addition, the detection of circRNA presence in the reaction mixture and validation of their circular nature are performed using specific primers in RT‐qPCR assays. To confirm the splice junctions of circRNAs, it is crucial to conduct Sanger sequencing on outward‐oriented PCR products (Figure 7B) [44].

### 6.2 CircRNA Purification and Quality Evaluation

Following preliminary data validation, RNA purification is performed using column extraction or HPLC (high performance liquid chromatography) to obtain highly purified circRNAs (Figure 7C) [91, 97]. The standard procedure for column extraction involves the enzymatic digestion of other impurities in reaction samples using RNase R and the excision of the gel band corresponding to circRNAs, followed by its dissolution. The RNase R‐digested reaction mixture can also be directly separated by using HPLC methods, such as size exclusion chromatography (SEC) and reversed‐phase HPLC (RP‐HPLC). Sometimes, phosphatase is also required to remove 5'‐PPP. Recently, Zhang et al. developed a novel multi‐step purification process that employs RNase R and phosphatase treatments to eliminate linear RNA and 5'‐PPP, as well as cellulose‐based filtration to selectively remove dsRNA [98]. These purification methods aim to minimize the activation of natural immune responses [49]. However, it is inevitable that achieving high purity will potentially increase the overall preparation cost of circRNAs. After purification, the purity of circRNAs is further assessed using capillary electrophoresis or other suitable techniques to meet the standards required for subsequent applications (Figure 7D) [91]. Overall, the implementation of these quality control and analysis techniques ensures the reliability and efficacy of circRNA for diverse applications.

## 7 Potential Applications of Coding circRNAs

Advancements in technology have partially addressed the challenges associated with circRNA identification and physiological function characterization, thereby facilitating a more comprehensive understanding of their cellular roles [99]. This includes elucidating their intrinsic functions such as acting as miRNA sponges, serving as anchors for circRNA binding proteins (cRBPs), regulating transcriptional processes, functioning as translational template, and operating as molecular switches in diverse cellular processes [100]. Based on these insights, endogenous circRNAs have emerged as promising targets for disease diagnosis and treatment, which has been comprehensively reviewed elsewhere [101]. Moreover, synthetic circRNAs in vitro have been developed and utilized within the biomedical field, particularly as a substitute for mRNA therapeutics. Herein, we highlight several representative therapeutic applications of circRNAs and elucidate the advantages and challenges that necessitate resolution in order to optimize their potential as therapeutics.

### 7.1 Vaccines

Vaccines are the most extensively investigated and clinically accepted application of mRNA‐based therapeutics. Following the remarkable efficacy demonstrated by mRNA vaccines against SARS‐CoV‐2 during the COVID‐19 pandemic [102], various new mRNA vaccine candidates are currently under development, encompassing influenza, cytomegalovirus (CMV), respiratory syncytial virus (RSV), varicella zoster virus (VZV), malaria, and cancer, etc. [103]. Compared to traditional vaccines, linear mRNA vaccines offer several advantages: (1) enhanced specificity and efficacy of immune responses mediated by B cells and T cells [103b]. (2) IVT enables facile large‐scale production in a cell‐free environment, facilitating rapid development, streamlined manufacturing processes, and cost‐effective production methods [104]. Moreover, for most emerging viral vaccines, the primary challenge lies not in the effectiveness of conventional approaches but rather in the need for expedited development and large‐scale deployment. The mRNA technology presents a promising avenue to address this new demand. (3) Safety advantages include no need for integration with the host genome and no DNA interaction, which eliminates risks associated with mutations within hosts [105]. However, in clinical practice, the frequency of systemic adverse events such as fever and fatigue is comparatively higher with COVID‐19 mRNA vaccines when compared to protein subunit vaccines and inactivated virus vaccines [106]. Therefore, it is imperative to conduct long‐term safety monitoring for COVID‐19 mRNA vaccines. Furthermore, due to the inherent instability of linear mRNA molecules, these vaccines necessitate ultra‐low temperature storage and cold chain transportation [107]. Even in high‐income countries, numerous clinics and vaccination sites struggle to meet the requirements for certain mRNA vaccines that mandate low‐temperature refrigeration. This challenge will be even more pronounced in low‐income countries. Consequently, there exists an urgent need for making improvements of these concerns.

**FIGURE 8:**
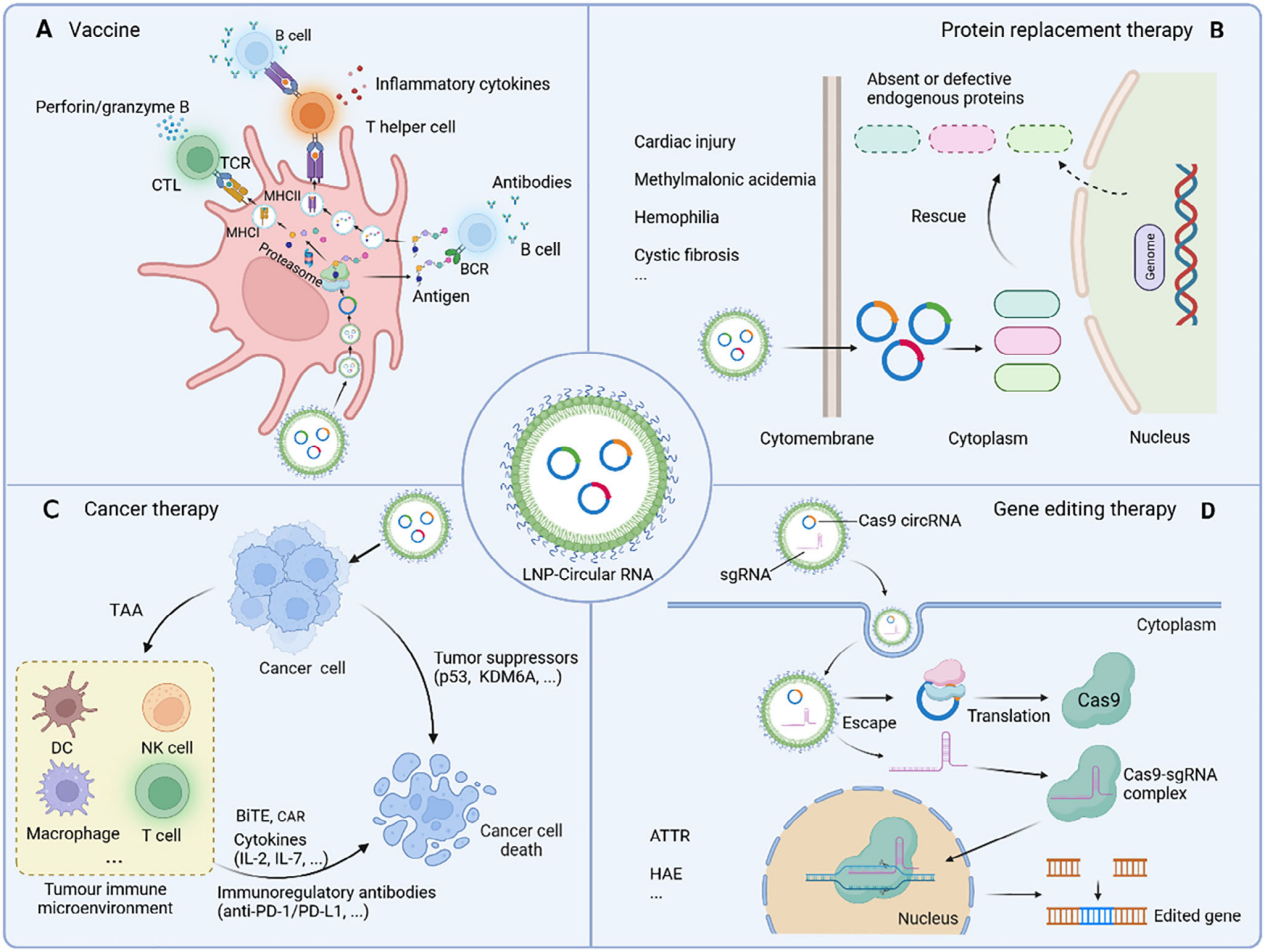
Potential application scenarios of protein‐coding circRNAs. (A) The circRNA vaccine is delivered into cells through LNP encapsulated or other strategies to induce both cytotoxic T cell (CTL) and B cell responses. (B) CircRNA therapy is an alternative method of proteotherapy to treat diseases through rescuing the absent or defective endogenous proteins. (C) CircRNAs are utilized to develop cancer therapeutics, such as encoding tumor suppressors (p53 or KDM6A), TAAs, immunostimulatory cytokines (IL‐2 and IL‐7), immunoregulatory antibodies (anti‐PD‐1/PD‐L1 or BiTE), or CARs for engineered T/NK/macrophage cell therapy. (D) The circRNA format of Cas9 is employed in the treatment of genetic diseases (e.g., ATTR and HAE) by correcting the causative mutations.

As a captivating alternative to mRNA, circRNA exhibits superior resistance to RNase and possesses significantly enhanced stability compared to mRNA. Previous studies have demonstrated that even after being stored at room temperature for a duration of 2 weeks, circRNA maintains both cell transfection efficiency and protein expression without any detrimental effects [7]. Moreover, circRNAs lack the 5'‐Cap and 3'‐polyA structures while obviating the need for modified bases during preparation. This simplifies the production process and reduces costs. These advantages underscore the potential application of circRNAs in vaccine development for emerging infectious diseases and cancers. A recent study by Qu et al. pioneered the development of a circRNA vaccine encoding the RNA‐binding domain (RBD) of the Spike protein against SARS‐CoV‐2 [7]. The authors demonstrated that, in comparison to the mRNA vaccine, this vaccine exhibited enhanced levels of antigen expression and prolonged antigen persistence, resulting in the generation of high titers of the neutralizing antibodies, Th1‐biased immune responses, and more efficacious preventive effects (Figure 8A) [7]. Similar research findings were also discovered in the study of the MPXV circRNA vaccine [108, 22b]. Another study conducted by Li et al. showcased that circRNAs‐based cancer vaccines could elicit robust T cell immune responses and effectively suppress tumor progression across various tumor models [22]. Notably, through synergistic effects with adoptive cell transfer therapy, the circRNAs vaccine even achieved complete regression of tumors in a late‐stage model [22].

These findings suggest that circRNA vaccines not only inherit the advantages of mRNA vaccine, but also have the potential to overcome the limitations associated with mRNA‐based vaccines. However, circRNA vaccines are still in the developmental stage, and prior to their successful clinical application, several key challenges need to be addressed. These include enhancing circularization efficiency, improving translation potency, and optimizing delivery systems for efficient and secure administration. For instance, a fundamental concern emerges as most RNA‐based vaccines mainly induce neutralizing IgG antibodies that provide protection against lower respiratory tract infections through systemic immunity. However, the induction of IgA antibodies, which are crucial for mucosal disinfection and immune defense in the upper respiratory tract, remains undetermined in current clinical or preclinical trials. Hence, there is an urgent need to develop novel delivery systems for effective mucosal vaccination against respiratory pathogens.

### 7.2 Protein Replacement Therapy

Following the tremendous success of RNA‐based vaccines, the exploration of utilizing mRNA to express therapeutic proteins has emerged as a pivotal objective in this field. Protein replacement therapies that are developed to reinstate the function of the absent or defective endogenous proteins stand out as a prominent avenue. The mRNAs encoding for functional endogenous proteins have been explored for several diseases, including cardiac injury, methylmalonic acidemia, hemophilia, cystic fibrosis and so on [109]. In contrast to mRNA vaccines, RNA‐based protein replacement therapies require overcoming additional obstacles. Particularly in enhancing protein expression levels and extending the durability of the therapy. Previous studies have demonstrated that mRNA protein replacement therapeutics require much higher (maybe 10‐1000‐fold) protein expression and more frequent dosing than vaccines for efficacy [2], while circRNAs which have higher cumulative protein production and sustained protein expression levels, should be a promising alternative (Figure 8B). Another advantage that makes circRNA more suitable for protein replacement therapies is its lower immunogenicity with higher redosing potential [21]. Orna Therapeutics company, established by Aderson et al., has reported their recent progress in treating Duchenne Muscular Dystrophy (DMD) at ASGCT 2022 through circRNA‐mediated expression of dystrophin, addressing a challenge posed by protein therapy due to the relatively high molecular weight of dystrophin. However, circRNA also faces similar challenges to linear mRNA, such as the need for precise delivery to target tissues beyond the liver. Addressing this issue necessitates further development and optimization of lipid nanoparticles and other delivery systems. Another concern is the precise regulation of circRNA expression levels to maintain appropriate functional levels without inducing toxicity due to overexpression. Additionally, although circRNAs exhibit lower immunogenicity, they can still activate the innate immune system. Further research should be conducted on circRNAs to enhance or regulate protein translation while minimizing the immunogenicity that caused adverse effects.

### 7.3 Cancer Therapy

Due to its remarkable flexibility and versatility, RNA‐based therapy has emerged as a potent modality for cancer treatment. RNA can be harnessed to encode tumor suppressors, genome editing proteins for precise tumor control, cancer neoantigens or tumor‐associated antigens (TAAs) as vaccines, immunostimulatory cytokines, immunoregulatory antibodies, bispecific T cell engager antibodies (BiTE) or chimeric antigen receptors (CAR) for engineered T/NK/macrophage cell therapy [109, 110]. Many of these therapeutic approaches have demonstrated promising efficacy in preclinical studies, with several progressing into clinical trials. The mRNA‐based cancer therapy has been comprehensively reviewed elsewhere [109i]. Here, we focus on circRNAs and highlight their advantages and existing crucial problems for cancer therapy. Several studies have shown that the expression of cytokines by circRNAs effectively modulates the intratumoral and systemic anti‐tumor immune responses, leading to significant tumor suppression (Figure 8C) [111]. In comparison to mRNA, circRNA exhibits remarkable stability, ensuring the prolonged expression of cytokines, resulting in effective recruitment and activation of immune cells in tumor tissues. Li et al. reported that circRNAs‐based cancer vaccines could elicit robust T cell immune responses and effectively suppress tumor progression across various tumor models [22a]. CircRNA‐based cancer vaccines ensure the consistent expression of antigens, promoting the prolonged antigen presentation and eliciting enhanced T cell immune responses [112]. Previous studies have also indicated that the lower immunogenicity of circRNA provides an advantage for therapeutic protein expression within tumors [8]. Additionally, compared with cap‐dependent translation, IRES‐based circRNA translation is less affected by cancer cell state and types, ensuring relatively consistent therapeutic protein expression and efficacy across different cancers [113].

Engineered immune cells, such as CAR‐T cells, CAR‐NK cells, and CAR‐macrophages, hold great promise as therapeutic modalities for cancer treatment [114]. Traditional manufacturing of immune cells has involved a complex and time‐consuming process encompassing the extraction of immune cells from the patient's blood, viral vector‐mediated transduction, and subsequent expansion to achieve sufficient cell numbers for infusion [115]. Alternatively, the utilization of mRNAs for the generation of CAR cells in vitro or in vivo through targeted delivery appears to be both feasible and effective [116]. For example, Rurik et al. demonstrated that anti‐FAP CAR T cells generated in vivo using CD5‐targeting lipid nanoparticles could efficiently eliminate fibrotic cells in the heart for the treatment of cardiac fibrosis [116a]. Other studies have also reported successful engineering of CAR‐T cells in vitro by electroporating mRNA for effective cancer treatment [116, 117]. However, it is important to note that mRNA translation reaches its peak within 24 h with a rapid decline thereafter [20]; therefore, these transient CAR cells must be administered or delivered quickly to ensure optimal CAR activity. While circRNAs hold great promise in CAR cell engineering due to their potent and stable protein expression abilities (Figure 8C) [89].

For RNA‐based cancer therapies, achieving optimal safety and efficacy necessitates the precise regulation of therapeutic protein expression and their targeted delivery to malignant tissues/cells, while minimizing potential off‐target effects on normal tissues/cells. For instance, prolonged production or excessive expression of immunostimulatory cytokines or immunoregulatory antibodies in circRNA therapeutics may heighten the risk of adverse side effects. The investigation of circRNA platforms incorporating on‐off or cell‐specific switches should be further pursued.

### 7.4 Gene Editing Therapy

Engineered nuclease‐based gene editing holds significant potential in the treatment of genetic diseases by correcting the causative mutations. Several nucleases have been adopted for gene editing therapy, including zinc finger nucleases (ZFNs), transcription activator‐like effector nucleases (TALENs), and RNA‐guided clustered regularly interspaced short palindromic repeats (CRISPR)/CRISPR‐associated (Cas) endonucleases [118]. Among these, CRISPR/Cas9 stands out as one of the most widely employed and straightforward systems for treating human genetic diseases, including cardiovascular diseases, neuro disorders, and cancers (Figure 8D) [119]. Additionally, CRISPR/Cas9‐derived base editing systems, prime editing systems, CRISPRi systems, and CRISPRa systems also show potential in treating diseases caused by abnormal gene expression [119, 120]. However, sustained expression of nucleases enhances the gene editing capabilities, while it also raises concerns about off‐target effects [121]. Alternatively, RNA‐encoded nucleases appear to be favorable options due to their transient expression and consequently resulting in less off‐target effects [122].

In 2022, Intellia Therapeutics reported the mid‐term clinical trial findings of its first in vivo CRISPR‐mRNA gene editing therapy, NTLA‐2001, for transthyretin amyloidosis (ATTR) [123]. The direct administration of CRISPR‐mRNA enables efficient intrabody gene editing, and a single dose resulted in sustained reduction of serum TTR protein levels for up to 12 months [124]. Additionally, Intellia Therapeutics disclosed positive mid‐term data from a phase 1/2 clinical trial of its second in vivo CRISPR‐mRNA gene editing therapy, NTLA‐2002, for the treatment of hereditary angioedema (HAE). Patients exhibited a significant decrease in disease frequency following a single intervention [125]. However, significant challenges also remain before CRISPR‐mRNA technology can be routinely employed in the clinic for treating genetic diseases. RNA and its encoded CRISPR/Cas components can elicit an immune response, leading to the elimination of edited cells [126]; hence, it is crucial for gene editing therapies utilizing RNA systems to possess minimal immunogenicity [127]. Consequently, circRNA with reduced immunogenicity emerges as a more suitable candidate compared to mRNA for gene editing therapy. While circRNA extends protein expression duration and enhances editing efficiency, it may also contribute to elevated off‐target effects. Moreover, CRISPR/Cas‐based gene editing proteins exhibit substantial size, with Cas9 itself reaching a size of 4 kb. However, current circulation methodologies still face great challenges in efficiently synthesizing circRNAs more than 5 kb. Therefore, further investigations are warranted to optimize the circulation strategy for large circRNA and mitigate off‐target risks while concurrently augmenting circRNA gene editing efficiency.

In addition to encoding nucleases, circRNA can also serve as a guide RNA (gRNA) to recruit Cas or its derived protein to enable more efficient programmable DNA or RNA editing. This is particularly advantageous since linear gRNA is highly susceptible to degradation by nucleases. Several studies have demonstrated that circularized gRNAs could significantly enhance both in vitro and in vivo DNA or RNA editing efficiency [128].

Compared to other RNA‐based therapies, gene editing therapy requires enhanced precision in targeted delivery, necessitating the precise delivery of gene editing components to specific tissues and cells for therapeutic efficacy [129]. While non‐viral vectors for mRNA and circRNA only enable limited tissue targeting, further advancements in delivery systems are imperative to precisely target specific organs and cells, thereby expanding the application range of RNA‐based gene editing therapy.

## 8 Conclusion and Future Perspectives

With the remarkable success of mRNA vaccines against SARS‐CoV‐2 during the COVID‐19 pandemic, mRNA therapeutics based on linear mRNA platform has emerged as a burgeoning field, witnessing a rapid surge in the number of clinical developments. The profound insights gained from the comprehensive development of COVID‐19 mRNA vaccine will be leveraged to optimize its application in diverse disease contexts, including cancer, autoimmunity, and genetic disorders. However, the clinical utility of linear mRNA therapeutics is still constrained by certain limitations. Linear mRNA necessitates extensive modifications to withstand RNases degradation and prevent innate immune stimulation [3], posing challenges in efficient manufacturing and incorporation into lipid nanoparticle carriers, thereby rendering the process costly. Despite these extensive modifications, linear mRNA remains relatively short‐lived, thereby restricting the production of therapeutic proteins per delivered molecule.

CircRNAs with covalently closed structures have emerged as a captivating alternative. In comparison to their linear mRNA counterparts, circRNAs exhibit prolonged half‐lives and stability, ameliorating the rigorous conditions for their storage and transportation [107]. In addition, prior investigations have demonstrated that unmodified circRNA is less immunogenic than unmodified linear mRNA within cellular systems [21]. Notably, transfection of circRNA resulted in significantly reduced release of cytokines, along with enhanced in vivo translation efficiency [21]. These improved pharmacokinetic profiles ultimately augment the in vivo production of therapeutic proteins, leading to a more potent therapeutic effect. Due to these advantages, the number of circRNA therapeutics under clinical development is growing rapidly, including vaccines, immunomodulatory antibodies, or cytokines for cancer, and in situ CAR‐T cell generation etc. However, several significant challenges remain unaddressed and necessitate further investigations in order to advance circRNA through all stages of pharmaceutical drug development towards commercialization for clinical applications in future researches.

Firstly, achieving sufficiently optimized circularization efficiency is crucial for circRNA synthesis, and it is imperative to dedicate more efforts towards developing strategies for this purpose. Group I intron‐based PIE systems have been extensively investigated, leading to the identification of multiple high‐efficiency circularization strategies at the lab‐scale. However, the successful scalability of these methods, particularly in large fragment RNA circularization while maintaining optimal circularization efficiency, requires further validation. Relying on our practical expertise, several factors including mRNA size, nucleotide sequences, and composition of the circularization buffer system should be meticulously considered and optimized to ensure enhanced RNA circularization efficiency.

The second potential concern arises from the possibility of an immune response triggered by circRNAs, potentially limiting its translation. The immunogenicity of circRNAs remains controversial to date. Autocatalytic splicing reactions during circRNA synthesis generate non‐circular byproducts, including linear RNA and baroque intron RNA. Therefore, one argument suggests that rigorous purification could render the immunogenicity negligible [21]. However, others argue that purification alone may not be sufficient to address this issue adequately. They emphasize that uncertainty regarding the ability of circRNA to trigger an immune response may stem from specific RNA sequences or structures, regardless of whether they are present in circRNAs or linear forms; both can still induce immunogenicity [44, 93]. Given this, drawing accurate conclusions may depend on the purity of circRNA samples, making it essential to minimize impurities in circRNAs. The HPLC method is relatively effective for circRNA separation in our lab‐scale studies; however, there is still room for improvement in terms of its precision and repeatability. Some novel separation technologies targeting circRNA purification represents a crucial direction in the field of circRNA therapeutics.

Additionally, the incorporation of poly(A), poly(AC) or other spacers in circRNAs to enhance their expression contradicts the diligent avoidance of introducing additional nucleotides at splicing junctions. Despite the frequent occurrence of ploy(A) in intracellular mature mRNA, the potential immunogenicity associated with these spacers in circRNAs remains largely unknown, as does their mechanism for enhancing circRNA translation. Therefore, it is beneficial to develop innovative alternative strategies, such as engineering or screening humanized IRES or IRES‐like elements with high‐activity [73], that can promote circRNA translation while minimizing immunogenicity.

Finally, developing safe and efficacious delivery vehicles poses a significant challenge in expanding the clinical application of RNA therapy. Currently, LNP serve as the most clinically advanced RNA delivery vehicles. However, emerging clinical studies have revealed that the presence of polyethylene glycol (PEG)‐modified lipids (key components of LNPs) can induce specific antibodies in circulation, potentially compromising therapeutic efficacy and leading to severe anaphylaxis and infusion reactions [130]. A recent study reported that pre‐existing anti‐PEG antibodies were detectable in plasma from 71% of subjects prior to mRNA vaccination [131]. The extensive utilization of PEGylated‐LNP based mRNA vaccines or RNA therapeutics exacerbates the situation, thereby necessitating the optimization of PEG‐modified lipids or exploration of alternative lipids as a prominent area of the next researches. Additionally, innovative delivery systems such as cell‐based approaches, extracellular vesicles, and bionic vesicles are currently undergoing preclinical studies for validation, potentially offering alternative methods of circRNA delivery.

In summary, circRNAs represent a burgeoning class of RNA therapeutics and we stand on the cusp of a new era in circRNA research and applications. Advanced techniques for synthesizing, purifying, and delivering circRNAs will be developed in the forthcoming years to expedite the progress of circRNA therapeutics. Despite existing challenges, circRNAs will comprehensively surpass mRNAs due to their unparalleled advantages in gene and cell therapy.

## Conflicts of Interest

The authors declare no conflicts of interest.

## References

[1] S. Qin , X. Tang , Y. Chen , et al., “mRNA‐Based Therapeutics: Powerful and Versatile Tools to Combat Diseases,” Signal Transduction and Targeted Therapy 7 (2022): 166. 35597779 10.1038/s41392-022-01007-w PMC9123296

[2] E. Rohner , R. Yang , K. S. Foo , A. Goedel , and K. R. Chien , “Unlocking the Promise of mRNA Therapeutics,” Nature Biotechnology 40 (2022): 1586–1600. 10.1038/s41587-022-01491-z 36329321

[3] S. C. Kim , S. S. Sekhon , W. R. Shin , et al., “Modifications of mRNA Vaccine Structural Elements for Improving mRNA Stability and Translation Efficiency,” Molecular and Cellular Toxicology 18 (2022): 1–8. 34567201 10.1007/s13273-021-00171-4 PMC8450916

[4] a) Y. Zong , Y. Lin , T. Wei , and Q. Cheng , “Lipid Nanoparticle (LNP) Enables mRNA Delivery for Cancer Therapy,” Advanced Materials 35, no. 51 (2023): e2303261. 37196221 10.1002/adma.202303261

[5] C. Schmidt and B. S. Schnierle , “Self‐Amplifying RNA Vaccine Candidates: Alternative Platforms for mRNA Vaccine Development,” Pathogens 12 (2023): 138. 10.3390/pathogens12010138 PMC9863218 36678486

[6] T. Beissert , M. Perkovic , A. Vogel , et al., “A Trans‐amplifying RNA Vaccine Strategy for Induction of Potent Protective Immunity,” Molecular Therapy 28 (2020): 119–128. 31624015 10.1016/j.ymthe.2019.09.009 PMC6953774

[7] a) L. Qu , Z. Yi , Y. Shen , et al., “Circular RNA Vaccines Against SARS‐CoV‐2 and Emerging Variants,” Cell 185 (2022): 1728–1744. 35460644 10.1016/j.cell.2022.03.044 PMC8971115

[8] T. Loan Young , K. Chang Wang , A. James Varley , and B. Li , “Clinical Delivery of Circular RNA: Lessons Learned From RNA Drug Development,” Advanced Drug Delivery Reviews 197 (2023): 114826. 37088404 10.1016/j.addr.2023.114826

[9] X. Li , L. Yang , and L. L. Chen , “The Biogenesis, Functions, and Challenges of Circular RNAs,” Molecular Cell 71 (2018): 428–442. 30057200 10.1016/j.molcel.2018.06.034

[10] H. L. Sanger , G. Klotz , D. Riesner , H. J. Gross , and A. K. Kleinschmidt , “Viroids are Single‐Stranded Covalently Closed Circular RNA Molecules Existing as Highly Base‐paired Rod‐Like Structures,” PNAS 73 (1976): 3852. 1069269 10.1073/pnas.73.11.3852 PMC431239

[11] a) W. Y. Zhou , Z. R. Cai , J. Liu , D. S. Wang , H. Q. Ju , and R. H. Xu , “Circular RNA: Metabolism, Functions and Interactions With Proteins,” Molecular Cancer 19 (2020): 172. 33317550 10.1186/s12943-020-01286-3 PMC7734744

[12] a) M. Lei , G. Zheng , Q. Ning , J. Zheng , and D. Dong , “Translation and Functional Roles of Circular RNAs in Human Cancer,” Molecular Cancer 19 (2020): 30. 32059672 10.1186/s12943-020-1135-7 PMC7023758

[13] J. O. Westholm , P. Miura , S. Olson , et al., “Genome‐wide Analysis of Drosophila Circular RNAs Reveals Their Structural and Sequence Properties and Age‐Dependent Neural Accumulation,” Cell Reports 9 (2014): 1966–1980. 25544350 10.1016/j.celrep.2014.10.062 PMC4279448

[14] A. Ivanov , S. Memczak , E. Wyler , et al., “Analysis of Intron Sequences Reveals Hallmarks of Circular RNA Biogenesis in Animals,” Cell Reports 10 (2015): 170–177. 25558066 10.1016/j.celrep.2014.12.019

[15] a) L. L. Chen , “The Expanding Regulatory Mechanisms and Cellular Functions of Circular RNAs,” Nature Reviews Molecular Cell Biology 21 (2020): 475–490. 32366901 10.1038/s41580-020-0243-y

[16] a) A. T. He , J. Liu , F. Li , and B. B. Yang , “Targeting Circular RNAs as a Therapeutic Approach: Current Strategies and Challenges,” Signal Transduction and Targeted Therapy 6 (2021): 185. 34016945 10.1038/s41392-021-00569-5 PMC8137869

[17] a) I. Legnini , G. Di Timoteo , F. Rossi , et al., “Circ‐ZNF609 Is a Circular RNA That Can Be Translated and Functions in Myogenesis,” Molecular Cell 66 (2017): 22–37. 28344082 10.1016/j.molcel.2017.02.017 PMC5387670

[18] a) B. D. Lee , U. Neri , S. Roux , et al., “Mining Metatranscriptomes Reveals a Vast World of Viroid‐Like Circular RNAs,” Cell 186 (2023): 646–661. 36696902 10.1016/j.cell.2022.12.039 PMC9911046

[19] a) J. Cheng , G. Li , W. Wang , D. B. Stovall , G. Sui , and D. Li , “Circular RNAs With Protein‐Coding Ability in Oncogenesis,” Biochimica et Biophysica Acta: Reviews on Cancer 1878 (2023): 188909. 37172651 10.1016/j.bbcan.2023.188909

[20] R. A. Wesselhoeft , P. S. Kowalski , and D. G. Anderson , “Engineering Circular RNA for Potent and Stable Translation in Eukaryotic Cells,” Nature Communications 9 (2018): 2629. 10.1038/s41467-018-05096-6 PMC6035260 29980667

[21] R. A. Wesselhoeft , P. S. Kowalski , F. C. Parker‐Hale , Y. Huang , N. Bisaria , and D. G. Anderson , “RNA Circularization Diminishes Immunogenicity and can Extend Translation Duration In Vivo,” Molecular Cell 74 (2019): 508–520. 30902547 10.1016/j.molcel.2019.02.015 PMC6724735

[22] a) H. Li , K. Peng , K. Yang , et al., “Circular RNA Cancer Vaccines Drive Immunity in Hard‐To‐Treat Malignancies,” Theranostics 12 (2022): 6422–6436. 36168634 10.7150/thno.77350 PMC9475446

[23] J. Li , D. Sun , W. Pu , J. Wang , and Y. Peng , “Circular RNAs in Cancer: Biogenesis, Function, and Clinical Significance,” Trends in Cancer 6 (2020): 319–336. 32209446 10.1016/j.trecan.2020.01.012

[24] a) Y. Zhang , X. O. Zhang , T. Chen , et al., “Circular Intronic Long Noncoding RNAs,” Molecular Cell 51 (2013): 792–806. 24035497 10.1016/j.molcel.2013.08.017

[25] J. U. Guo , V. Agarwal , H. Guo , and D. P. Bartel , “Expanded Identification and Characterization of Mammalian Circular RNAs,” Genome Biology 15 (2014): 409. 25070500 10.1186/s13059-014-0409-z PMC4165365

[26] a) L. L. Chen and L. Yang , “Regulation of circRNA Biogenesis,” RNA Biology 12 (2015): 381–388. 25746834 10.1080/15476286.2015.1020271 PMC4615371

[27] C. Yan , R. Wan , and Y. Shi , “Molecular Mechanisms of Pre‐mRNA Splicing Through Structural Biology of the Spliceosome,” Cold Spring Harbor Perspectives in Biology 11 (2019): a032409. 30602541 10.1101/cshperspect.a032409 PMC6314064

[28] B. Xu , Y. Meng , and Y. Jin , “RNA Structures in Alternative Splicing and Back‐Splicing,” Wiley Interdisciplinary Reviews: RNA 12 (2021): e1626. 32929887 10.1002/wrna.1626

[29] a) L. L. Chen , “The Biogenesis and Emerging Roles of Circular RNAs,” Nature Reviews Molecular Cell Biology 17 (2016): 205–211. 10.1038/nrm.2015.32 26908011

[30] a) R. Ashwal‐Fluss , M. Meyer , N. R. Pamudurti , et al., “circRNA Biogenesis Competes With Pre‐mRNA Splicing,” Molecular Cell 56 (2014): 55–66. 25242144 10.1016/j.molcel.2014.08.019

[31] S. P. Barrett , P. L. Wang , and J. Salzman , “Circular RNA Biogenesis can Proceed Through an Exon‐Containing Lariat Precursor,” Elife 4 (2015): e07540. 26057830 10.7554/eLife.07540 PMC4479058

[32] A. Robic and C. Kuhn , “Beyond Back Splicing, a Still Poorly Explored World: Non‐Canonical Circular RNAs,” Genes (Basel) 11 (2020): 1111. 32972011 10.3390/genes11091111 PMC7565381

[33] Z. Lu , G. S. Filonov , J. J. Noto , et al., “Metazoan tRNA Introns Generate Stable Circular RNAs In Vivo,” RNA 21 (2015): 1554–1565. 26194134 10.1261/rna.052944.115 PMC4536317

[34] S. Petkovic and S. Muller , “RNA Circularization Strategies In Vivo and In Vitro,” Nucleic Acids Research 43 (2015): 2454–2465. 25662225 10.1093/nar/gkv045 PMC4344496

[35] R. Flores , D. Grubb , A. Elleuch , M. A. Nohales , S. Delgado , and S. Gago , “Rolling‐Circle Replication of Viroids, Viroid‐Like Satellite RNAs and Hepatitis Delta Virus: Variations on a Theme,” RNA Biology 8 (2011): 200–206. 21358283 10.4161/rna.8.2.14238

[36] a) D. C. Tatomer , D. Liang , and J. E. Wilusz , “Inducible Expression of Eukaryotic Circular RNAs From Plasmids,” Methods in Molecular Biology 1648 (2017): 143–154. 28766295 10.1007/978-1-4939-7204-3_11

[37] P. Obi and Y. G. Chen , “The Design and Synthesis of Circular RNAs,” Methods 196 (2021): 85–103. 33662562 10.1016/j.ymeth.2021.02.020 PMC8670866

[38] B. Beckert and B. Masquida , “Synthesis of RNA by In Vitro Transcription,” Methods in Molecular Biology 703 (2011): 29–41. 21125481 10.1007/978-1-59745-248-9_3

[39] a) N. G. Dolinnaya , N. I. Sokolova , D. T. Ashirbekova , and Z. A. Shabarova , “The Use of BrCN for Assembling Modified DNA Duplexes and DNA‐RNA Hybrids; Comparison With Water‐Soluble Carbodiimide,” Nucleic Acids Research 19 (1991): 3067–3072. 1711679 10.1093/nar/19.11.3067 PMC328272

[40] S. Petkovic and S. Muller , “Synthesis and Engineering of Circular RNAs,” Methods in Molecular Biology 1724 (2018): 167–180. 29322449 10.1007/978-1-4939-7562-4_14

[41] X. Chen and Y. Lu , “Circular RNA: Biosynthesis In Vitro,” Frontiers in Bioengineering and Biotechnology 9 (2021): 787881. 34917603 10.3389/fbioe.2021.787881 PMC8670002

[42] N. Abe , A. Kodama , and H. Abe , “Circular RNAs, methods and protocols – preparation of circRNA in vitro,” Methods in Molecular Biology 1724 (2018): 181–192. 29322450 10.1007/978-1-4939-7562-4_15

[43] M. J. Moore and C. C. Query , “Joining of RNAs by Splinted Ligation,” Methods in Enzymology 317 (2000): 109–123. 10829275 10.1016/s0076-6879(00)17009-0

[44] E. M. Carmona , “Circular RNA: Design Criteria for Optimal Therapeutical Utility” (PhD diss., Harvard University, 2019).

[45] S. Muller and B. Appel , “In Vitro Circularization of RNA,” RNA Biology 14 (2017): 1018–1027. 27668458 10.1080/15476286.2016.1239009 PMC5680678

[46] a) J. Nandakumar , C. K. Ho , C. D. Lima , and S. Shuman , “RNA Substrate Specificity and Structure‐Guided Mutational Analysis of Bacteriophage T4 RNA Ligase 2,” Journal of Biological Chemistry 279 (2004): 31337–31347. 15084599 10.1074/jbc.M402394200

[47] a) S. Petkovic and S. Muller , “RNA Self‐Processing: Formation of Cyclic Species and Concatemers From a Small Engineered RNA,” FEBS Letters 587 (2013): 2435–2440. 23796421 10.1016/j.febslet.2013.06.013

[48] A. R. Ferre‐D'Amare and W. G. Scott , “Small Self‐Cleaving Ribozymes,” Cold Spring Harbor Perspectives in Biology 2 (2010): a003574. 20843979 10.1101/cshperspect.a003574 PMC2944367

[49] C. Chen , H. Wei , K. Zhang , et al., “A Flexible, Efficient, and Scalable Platform to Produce Circular RNAs as New Therapeutics,” preprint, bioRxiv 494115, May 31, 2022, 10.1101/2022.05.31.494115.

[50] R. M. Jimenez , J. A. Polanco , and A. Luptak , “Chemistry and Biology of Self‐Cleaving Ribozymes,” Trends in Biochemical Sciences 40 (2015): 648–661. 26481500 10.1016/j.tibs.2015.09.001 PMC4630146

[51] A. M. Diegelman and E. T. Kool , “Generation of Circular RNAs and Trans‐Cleaving Catalytic RNAs by Rolling Transcription of Circular DNA Oligonucleotides Encoding Hairpin Ribozymes,” Nucleic Acids Research 26 (1998): 3235–3241. 9628924 10.1093/nar/26.13.3235 PMC147673

[52] a) S. M. Nesbitt , H. A. Erlacher , and M. J. Fedor , “The Internal Equilibrium of the Hairpin Ribozyme: Temperature, Ion and pH Effects,” Journal of Molecular Biology 286 (1999): 1009–1024. 10047478 10.1006/jmbi.1999.2543

[53] P. Haugen , D. M. Simon , and D. Bhattacharya , “The Natural History of Group I Introns,” Trends in Genetics 21 (2005): 111–119. 15661357 10.1016/j.tig.2004.12.007

[54] H. Nielsen and S. D. Johansen , “Group I Introns: Moving in New Directions,” RNA Biology 6 (2009): 375–383. 19667762 10.4161/rna.6.4.9334

[55] D. Bhattacharya , V. Reeb , D. M. Simon , and F. Lutzoni , “Phylogenetic Analyses Suggest Reverse Splicing Spread of Group I Introns in Fungal Ribosomal DNA,” BMC Evolutionary Biology 5 (2005): 68. 16300679 10.1186/1471-2148-5-68 PMC1299323

[56] M. Felletti and J. S. Hartig , “Ligand‐Dependent Ribozymes,” Wiley Interdisciplinary Reviews: RNA 8 (2017): e1395. 10.1002/wrna.1395 27687155

[57] J. W. Rausch , W. F. Heinz , M. J. Payea , C. Sherpa , M. Gorospe , and S. F. J. Le Grice , “Characterizing and Circumventing Sequence Restrictions for Synthesis of Circular RNA In Vitro,” Nucleic Acids Research 49 (2021): e35. 33406226 10.1093/nar/gkaa1256 PMC8034654

[58] M. Puttaraju and M. D. Been , “Group I Permuted Intron‐exon (PIE) Sequences Self‐splice to Produce Circular Exons,” Nucleic Acids Research 20 (1992): 5357–5364. 1279519 10.1093/nar/20.20.5357 PMC334342

[59] E. Ford , “Synthesis of Circular RNA in Bacteria and Yeast Using RNA Cyclase Ribozymes Derived From a Group I Intron of Phage T4,” PNAS 91 (1994): 3117–3121. 7512723 10.1073/pnas.91.8.3117 PMC43526

[60] A. M. Lambowitz and S. Zimmerly , “Group II Introns: Mobile Ribozymes That Invade DNA,” Cold Spring Harbor Perspectives in Biology 3 (2011): a003616. 20463000 10.1101/cshperspect.a003616 PMC3140690

[61] a) A. M. Pyle , “The Tertiary Structure of Group II Introns: Implications for Biological Function and Evolution,” Critical Reviews in Biochemistry and Molecular Biology 45 (2010): 215–232. 20446804 10.3109/10409231003796523 PMC4408542

[62] A. M. Pyle , “Group II Intron Self‐Splicing,” Annual Review of Biophysics 45 (2016): 183. 10.1146/annurev-biophys-062215-011149 27391926

[63] A. Barkan , Molecular Biology and Biotechnology of Plant Organelles: Chloroplasts and Mitochondria. (Springer, 2004).

[64] R. T. Chan , J. K. Peters , A. R. Robart , T. Wiryaman , K. R. Rajashankar , and N. Toor , “Structural Basis for the Second Step of Group II Intron Splicing,” Nature Communications 9 (2018): 4676. 10.1038/s41467-018-06678-0 PMC6224600 30410046

[65] R. Perriman and M. Ares , “Circular mRNA Can Direct Translation of Extremely Long Repeating‐sequence Proteins In Vivo,” RNA 4 (1998): 1047–1054. 9740124 10.1017/s135583829898061x PMC1369681

[66] a) N. Abe , M. Hiroshima , H. Maruyama , et al., “Rolling Circle Amplification in a Prokaryotic Translation System Using Small Circular RNA,” Angewandte Chemie International Edition in English 52 (2013): 7004–7008. 10.1002/anie.201302044 23716491

[67] X. Fan , Y. Yang , C. Chen , and Z. Wang , “Pervasive Translation of Circular RNAs Driven by Short IRES‐Like Elements,” Nature Communications 13 (2022): 3751. 10.1038/s41467-022-31327-y PMC9242994 35768398

[68] a) L. J. Deng , W. Q. Deng , S. R. Fan , et al., “m6A Modification: Recent Advances, Anticancer Targeted Drug Discovery and Beyond,” Molecular Cancer 21 (2022): 52. 35164788 10.1186/s12943-022-01510-2 PMC8842557

[69] M. Imanishi , “Mechanisms and Strategies for Determining m6A RNA Modification Sites by Natural and Engineered m6A Effector Proteins,” Chemistry ‐ An Asian Journal 17 (2022): e202200367. 35750635 10.1002/asia.202200367

[70] a) J. Liu , Y. Yue , D. Han , et al., “A METTL3–METTL14 Complex Mediates Mammalian Nuclear RNA N6‐Adenosine Methylation,” Nature Chemical Biology 10 (2014): 93–95. 24316715 10.1038/nchembio.1432 PMC3911877

[71] a) Y. Yang , X. Fan , M. Mao , et al., “Extensive Translation of Circular RNAs Driven by N6‐Methyladenosine,” Cell Research 27 (2017): 626–641. 28281539 10.1038/cr.2017.31 PMC5520850

[72] Y. Shi , X. Jia , and J. Xu , “The New Function of circRNA: Translation,” Clinical and Translational Oncology 22 (2020): 2162–2169. 32449127 10.1007/s12094-020-02371-1

[73] R. Chen , S. K. Wang , J. A. Belk , et al., “Engineering Circular RNA for Enhanced Protein Production,” Nature Biotechnology 41 (2023): 262–272. 10.1038/s41587-022-01393-0 PMC9931579 35851375

[74] a) Y. G. Chen , R. Chen , S. Ahmad , et al., “N6‐Methyladenosine Modification Controls Circular RNA Immunity,” Molecular Cell 76 (2019): 96–109. 31474572 10.1016/j.molcel.2019.07.016 PMC6778039

[75] a) S. K. Jang , H. G. Krausslich , M. J. Nicklin , G. M. Duke , A. C. Palmenberg , and E. Wimmer , “A Segment of the 5' Nontranslated Region of Encephalomyocarditis Virus RNA Directs Internal Entry of Ribosomes During in Vitro Translation,” Journal of Virology 62 (1988): 2636–2643. 2839690 10.1128/jvi.62.8.2636-2643.1988 PMC253694

[76] a) S. D. Baird , M. Turcotte , R. G. Korneluk , and M. Holcik , “Searching for IRES,” RNA 12 (2006): 1755–1785. 16957278 10.1261/rna.157806 PMC1581980

[77] F. Buttgereit and M. D. Brand , “A Hierarchy of ATP‐Consuming Processes in Mammalian Cells,” Biochemical Journal 312, (1995): 163–167. 7492307 10.1042/bj3120163 PMC1136240

[78] a) A. Sriram , J. Bohlen , and A. A. Teleman , “Translation Acrobatics: How Cancer Cells Exploit Alternate Modes of Translational Initiation,” EMBO Reports 19 (2018): e45947. 30224410 10.15252/embr.201845947 PMC6172470

[79] a) Y. Yang and Z. Wang , “IRES‐Mediated Cap‐Independent Translation, A Path Leading to Hidden Proteome,” Journal of Molecular Cell Biology 11 (2019): 911–919. 31504667 10.1093/jmcb/mjz091 PMC6884710

[80] M. Tusup , T. Kundig , and S. Pascolo , “An eIF4G‐Recruiting Aptamer Increases the Functionality of In Vitro Transcribed mRNA,” EPH‐International Journal of Medical and Health Science 3 (2017): 20–25.

[81] a) Y. Kanamori and N. Nakashima , “A Tertiary Structure Model of the Internal Ribosome Entry Site (IRES) for Methionine‐Independent Initiation of Translation,” RNA 7 (2001): 266–274. 11233983 10.1017/s1355838201001741 PMC1370084

[82] a) C. M. Spahn , E. Jan , A. Mulder , R. A. Grassucci , P. Sarnow , and J. Frank , “Cryo‐EM Visualization of a Viral Internal Ribosome Entry Site Bound to Human Ribosomes,” Cell 118 (2004): 465–475. 15315759 10.1016/j.cell.2004.08.001

[83] A. V. Pisarev , L. S. Chard , Y. Kaku , H. L. Johns , I. N. Shatsky , and G. J. Belsham , “Functional and Structural Similarities Between the Internal Ribosome Entry Sites of Hepatitis C Virus and Porcine Teschovirus, a Picornavirus,” Journal of Virology 78 (2004): 4487–4497. 15078929 10.1128/JVI.78.9.4487-4497.2004 PMC387690

[84] a) K. M. Lee , C. J. Chen , and S. R. Shih , “Regulation Mechanisms of Viral IRES‐Driven Translation,” Trends in Microbiology 25 (2017): 546–561. 28242053 10.1016/j.tim.2017.01.010

[85] A. Koch , L. Aguilera , T. Morisaki , B. Munsky , and T. J. Stasevich , “Quantifying the Dynamics of IRES and Cap Translation With Single‐Molecule Resolution in Live Cells,” Nature Structural and Molecular Biology 27 (2020): 1095–1104. 10.1038/s41594-020-0504-7 PMC8351040 32958947

[86] H. Chen , D. Liu , A. Aditham , et al., “Chemical and Topological Design of Multicapped mRNA and Capped Circular RNA to Augment Translation,” Nature Biotechnology, (2024): 10.1038/s41587‐024‐02393‐y. 10.1038/s41587-024-02393-y PMC11929619 39313647

[87] K. Fukuchi , Y. Nakashima , N. Abe , et al., “Internal Cap‐Initiated Translation for Efficient Protein Production From Circular mRNA,” Nature Biotechnology (2025): 10.1038/s41587‐025‐02561‐8. 10.1038/s41587-025-02561-8 39972222

[88] X. Zhao , Y. Zhong , X. Wang , J. Shen , and W. An , “Advances in Circular RNA and Its Applications,” International Journal of Medical Sciences 19 (2022): 975–985. 35813288 10.7150/ijms.71840 PMC9254372

[89] K. Garber , “Orna Therapeutics: Circular Logic,” Nature Biotechnology (2022): 10.1038/d41587‐022‐00005‐1. 10.1038/d41587-022-00005-1 35931823

[90] a) A. Aditham , H. Shi , J. Guo , et al., “Chemically Modified mocRNAs for Highly Efficient Protein Expression in Mammalian Cells,” ACS Chemical Biology 17 (2022): 3352–3366. 34995053 10.1021/acschembio.1c00569

[91] Z. Qiu , Q. Hou , Y. Zhao , et al., “Clean‐PIE: A Novel Strategy for Efficiently Constructing Precise circRNA With Thoroughly Minimized Immunogenicity to Direct Potent and Durable Protein Expression” preprint, bioRxiv 496777, June 20, 2022, 10.1101/2022.06.20.496777.

[92] K. H. Lee , S. Kim , J. Song , S. R. Han , J. H. Kim , and S.‐W. Lee , “Efficient Circular RNA Engineering by End‐To‐End Self‐Targeting and Splicing Reaction Using Tetrahymena Group I Intron Ribozyme,” Molecular Therapy Nucleic Acids 33 (2023): 587–598. 37637208 10.1016/j.omtn.2023.07.034 PMC10457212

[93] C. X. Liu , S. K. Guo , F. Nan , Y. F. Xu , L. Yang , and L. L. Chen , “RNA Circles With Minimized Immunogenicity as Potent PKR Inhibitors,” Molecular Cell 82 (2022): 420–434. 34951963 10.1016/j.molcel.2021.11.019

[94] S. Qi , H. Wang , G. Liu , Q. Qin , P. Gao , and B. Ying , “Efficient Circularization of Protein‐Encoding RNAs Via a Novel Cis‐Splicing System,” Nucleic Acids Research 52 (2024): 10400–10415. 39162233 10.1093/nar/gkae711 PMC11417360

[95] C. I. Su , Z. S. Chuang , C. T. Shie , H. I. Wang , Y. T. Kao , and C. Y. Yu , “A Cis‐Acting Ligase Ribozyme Generates Circular RNA In Vitro for Ectopic Protein Functioning,” Nature Communications 15 (2024): 6607. 10.1038/s41467-024-51044-y PMC11298514 39098891

[96] J. Breuer , P. Barth , Y. Noe , et al., “What Goes Around Comes Around: Artificial Circular RNAs Bypass Cellular Antiviral Responses,” Molecular Therapy Nucleic Acids 28 (2022): 623–635. 35497503 10.1016/j.omtn.2022.04.017 PMC9042720

[97] B. T. Abe , R. A. Wesselhoeft , R. Chen , D. G. Anderson , and H. Y. Chang , “Circular RNA Migration in Agarose Gel Electrophoresis,” Molecular Cell 82 (2022): 1768–1777. 35358469 10.1016/j.molcel.2022.03.008 PMC9081248

[98] Z. Zhang , W. Li , X. Ren , et al., “Mitigating Cellular Dysfunction Through Contaminant Reduction in Synthetic circRNA for High‐Efficiency mRNA‐Based Cell Reprogramming,” Advanced Science 12 (2025): e2416629. 40042035 10.1002/advs.202416629 PMC12021033

[99] X. Meng , Q. Chen , P. Zhang , and M. Chen , “CircPro: An Integrated Tool for the Identification of circRNAs With Protein‐Coding Potential,” Bioinformatics 33 (2017): 3314–3316. 29028266 10.1093/bioinformatics/btx446

[100] M. Lu , “Circular RNA: Functions, Applications and Prospects,” ExRNA 2 (2020): 1.

[101] C. X. Liu and L. L. Chen , “Circular RNAs: Characterization, Cellular Roles, and Applications,” Cell 185 (2022): 2390. 35750036 10.1016/j.cell.2022.06.001

[102] a) N. Khehra , I. Padda , U. Jaferi , H. Atwal , S. Narain , and M. S. Parmar , “Tozinameran (BNT162b2) Vaccine: The Journey From Preclinical Research to Clinical Trials and Authorization,” Aaps Pharmscitech 22 (2021): 172. 34100150 10.1208/s12249-021-02058-y PMC8184133

[103] a) J. A. Whitaker , H. M. E. Sahly , and C. M. Healy , “mRNA Vaccines Against Respiratory Viruses,” Current Opinion in Infectious Diseases 36 (2023): 385–393. 37462930 10.1097/QCO.0000000000000948

[104] P. Rzymski , A. Szuster‐Ciesielska , T. Dzieciatkowski , W. Gwenzi , and A. Fal , “mRNA Vaccines: The Future of Prevention of Viral Infections?,” Journal of Medical Virology 95 (2023): e28572. 36762592 10.1002/jmv.28572

[105] F. Oguz and H. Atmaca , “mRNA as a Therapeutics: Understanding mRNA Vaccines,” Advanced Pharmaceutical Bulletin 12 (2022): 274–282. 35620336 10.34172/apb.2022.028 PMC9106950

[106] Q. Wu , M. Z. Dudley , X. Chen , et al., “Evaluation of the Safety Profile of COVID‐19 Vaccines: A Rapid Review,” BMC Medicine 19 (2021): 173. 34315454 10.1186/s12916-021-02059-5 PMC8315897

[107] M. N. Uddin and M. A. Roni , “Challenges of Storage and Stability of mRNA‐Based COVID‐19 Vaccines,” Vaccines (Basel) 9 (2021): 1033. 34579270 10.3390/vaccines9091033 PMC8473088

[108] Y. Sang , Z. Zhang , F. Liu , et al., “Monkeypox Virus Quadrivalent mRNA Vaccine Induces Immune Response and Protects Against Vaccinia Virus,” Signal Transduction and Targeted Therapy 8 (2023): 172. 10.1038/s41392-023-01432-5 PMC10144886 37117161

[109] a) A. Magadum , “Modified mRNA Therapeutics for Heart Diseases,” International Journal of Molecular Sciences 23 (2022): 15514. 36555159 10.3390/ijms232415514 PMC9779737

[110] a) X. Liu , D. M. Barrett , S. Jiang , et al., “Improved Anti‐Leukemia Activities of Adoptively Transferred T Cells Expressing Bispecific T‐Cell Engager in Mice,” Blood Cancer Journal no. 6 (2016): e430. 27258611 10.1038/bcj.2016.38 PMC5141353

[111] a) J. Yang , J. Zhu , J. Sun , et al., “Intratumoral Delivered Novel Circular mRNA Encoding Cytokines for Immune Modulation and Cancer Therapy,” Molecular Therapy Nucleic Acids 30 (2022): 184–197. 36156907 10.1016/j.omtn.2022.09.010 PMC9482165

[112] Y. Zhang , X. Liu , T. Shen , et al., “Small Circular RNAs as Vaccines for Cancer Immunotherapy,” Nature Biomedical Engineering 9 (2025): 249–267. 10.1038/s41551-025-01344-5 PMC12100636 39920212

[113] N. Sonenberg and A. G. Hinnebusch , “Regulation of Translation Initiation in Eukaryotes: Mechanisms and Biological Targets,” Cell 136 (2009): 731–745. 19239892 10.1016/j.cell.2009.01.042 PMC3610329

[114] K. Pan , H. Farrukh , V. Chittepu , H. Xu , C. X. Pan , and Z. Zhu , “CAR Race to Cancer Immunotherapy: From CAR T, CAR NK to CAR Macrophage Therapy,” Journal of Experimental and Clinical Cancer Research 41 (2022): 119. 35361234 10.1186/s13046-022-02327-z PMC8969382

[115] M. P. Jogalekar , R. L. Rajendran , F. Khan , C. Dmello , P. Gangadaran , and B. C. Ahn , “CAR T‐Cell‐Based Gene Therapy for Cancers: New Perspectives, Challenges, and Clinical Developments,” Frontiers in Immunology 13 (2022): 925985. 35936003 10.3389/fimmu.2022.925985 PMC9355792

[116] a) J. G. Rurik , I. Tombacz , A. Yadegari , et al., “CAR T Cells Produced In Vivo to Treat Cardiac Injury,” Science 375 (2022): 91–96. 34990237 10.1126/science.abm0594 PMC9983611

[117] M. M. Billingsley , N. Singh , P. Ravikumar , R. Zhang , C. H. June , and M. J. Mitchell , “Ionizable Lipid Nanoparticle‐Mediated mRNA Delivery for Human CAR T Cell Engineering,” Nano Letters 20 (2020): 1578–1589. 31951421 10.1021/acs.nanolett.9b04246 PMC7313236

[118] a) M. P. Hirakawa , R. Krishnakumar , J. A. Timlin , J. P. Carney , and K. S. Butler , “Gene Editing and CRISPR in the Clinic: Current and Future Perspectives,” Bioscience Reports 40, (2020): BSR20200127. 32207531 10.1042/BSR20200127 PMC7146048

[119] a) J. A. Doudna , “The Promise and Challenge of Therapeutic Genome Editing,” Nature 578 (2020): 229–236. 32051598 10.1038/s41586-020-1978-5 PMC8992613

[120] a) L. S. Qi , M. H. Larson , L. A. Gilbert , et al., “Repurposing CRISPR as an RNA‐Guided Platform for Sequence‐Specific Control of Gene Expression,” Cell 184 (2021): 844. 33545038 10.1016/j.cell.2021.01.019

[121] a) M. Chen , A. Mao , M. Xu , Q. Weng , J. Mao , and J. Ji , “CRISPR‐Cas9 for Cancer Therapy: Opportunities and Challenges,” Cancer Letters 447 (2019): 48–55. 30684591 10.1016/j.canlet.2019.01.017

[122] H. X. Zhang , Y. Zhang , and H. Yin , “Genome Editing With mRNA Encoding ZFN, TALEN, and Cas9,” Molecular Therapy 27 (2019): 735–746. 30803822 10.1016/j.ymthe.2019.01.014 PMC6453514

[123] K. Wood , M. Pink , and J. Seitzer , “Development of NTLA‐2001, a CRISPR/Cas9 Genome Editing Therapeutic for the Treatment of ATTR,” paper presented at the Second European Congress for ATTR Amyloidosis, Berlin, September 1–3, 2019.

[124] J. D. Gillmore , E. Gane , J. Taubel , et al., “CRISPR‐Cas9 In Vivo Gene Editing for Transthyretin Amyloidosis,” New England Journal of Medicine 385 (2021): 493–502. 34215024 10.1056/NEJMoa2107454

[125] *Intellia Therapeutics: Press Release* (Annual Scientific Meeting of the American College of Allergy, Asthma and Immunology, 2022).

[126] a) I. Hoerr , R. Obst , H. G. Rammensee , and G. Jung , “In Vivo Application of RNA Leads to Induction of Specific Cytotoxic T Lymphocytes and Antibodies,” European Journal of Immunology 30 (2000): 1–7. 10602021 10.1002/1521-4141(200001)30:1<1::AID-IMMU1>3.0.CO;2-#

[127] M. Tong , N. Palmer , A. Dailamy , et al., “Robust Genome and Cell Engineering via In Vitro and In Situ Circularized RNAs,” Nature Biomedical Engineering 9 (2025): 109–126. 10.1038/s41551-024-01245-z PMC12186994 39187662

[128] a) Z. Yi , L. Qu , H. Tang , et al., “Engineered Circular ADAR‐Recruiting RNAs Increase the Efficiency and Fidelity of RNA Editing In Vitro and In Vivo,” Nature Biotechnology 40 (2022): 946–955. 10.1038/s41587-021-01180-3 35145313

[129] Z. Xu , Q. Wang , H. Zhong , et al., “Carrier Strategies Boost the Application of CRISPR/Cas System in Gene Therapy,” Exploration (Beijing) 2 (2022): 20210081. 37323878 10.1002/EXP.20210081 PMC10190933

[130] D. D. Kang , X. Hou , L. Wang , et al., “Engineering LNPs With Polysarcosine Lipids for mRNA Delivery,” Bioactive Materials 37 (2024): 86–93. 38523704 10.1016/j.bioactmat.2024.03.017 PMC10957522

[131] Y. Ju , W. S. Lee , E. H. Pilkington , et al., “Anti‐PEG Antibodies Boosted in Humans by SARS‐CoV‐2 Lipid Nanoparticle mRNA Vaccine,” ACS Nano 16 (2022): 11769–11780. 35758934 10.1021/acsnano.2c04543

